# A one-dimensional conductive metal-organic framework with extended *π-d* conjugated nanoribbon layers

**DOI:** 10.1038/s41467-022-35315-0

**Published:** 2022-12-09

**Authors:** Shengcong Shang, Changsheng Du, Youxing Liu, Minghui Liu, Xinyu Wang, Wenqiang Gao, Ye Zou, Jichen Dong, Yunqi Liu, Jianyi Chen

**Affiliations:** 1 Beijing National Laboratory for Molecular Sciences, Key Laboratory of Organic Solids, Institute of Chemistry, Chinese Academy of Sciences, 100190 Beijing, P. R. China; 2 University of Chinese Academy of Sciences, 100049 Beijing, P. R. China

**Keywords:** Ligands, Electronic properties and materials, Electronic devices

## Abstract

Conductive metal-organic frameworks (MOFs) have performed well in the fields of energy and catalysis, among which two-dimensional (2D) and three-dimensional (3D) MOFs are well-known. Here, we have synthesized a one-dimensional (1D) conductive metal-organic framework (MOF) in which hexacoordinated 1,5-Diamino-4,8-dihydroxy-9,10-anthraceneedione (DDA) ligands are connected by double Cu ions, resulting in nanoribbon layers with 1D *π-d* conjugated nanoribbon plane and out-of-plane *π-π* stacking, which facilitates charge transport along two dimensions. The DDA-Cu as a highly conductive n-type MOF has high crystalline quality with a conductivity of ~ 9.4 S·m^−1^, which is at least two orders of magnitude higher than that of conventional 1D MOFs. Its electrical band gap (E_g_) and exciton binding energy (E_b_) are approximately 0.49 eV and 0.3 eV, respectively. When utilized as electrode material in a supercapacitor, the DDA-Cu exhibits good charge storage capacity and cycle stability. Meanwhile, as thse active semiconductor layer, it successfully simulates the artificial visual perception system with excellent bending resistance and air stability as a MOF-based flexible optoelectronic synaptic case. The controllable preparation of high-quality 1D DDA-Cu MOF may enable new architectural designs and various applications in the future.

## Abstract (web summary)

2D and 3D conductive MOFs have performed well in the fields of energy and catalysis. Here, authors synthesise a 1D conductive MOF in which DDA ligands are connected by double Cu ions, forming nanoribbon layers with π-d conjugated nanoribbon planes and out-of-plane π-π stacking, which facilitates charge transport along two dimensions.

## Introduction

One-dimensional (1D) materials such as nanofibers^1^, nanorods^2,3^, nanotubes^4,5^, and nanoribbons^6–8^ are an integral part of our technological society, which are widely utilized in several applications, including electronics, supercapacitors, membrane, catalysis, and sensors. In particular, inorganic 1D materials based on ZnO and carbon nanotubes have been intensively studied^9–11^. Graphene is a two-dimensional (2D) nanomaterial^12^. However, its nanoribbon allotrope constructed via covalent chemistry is a typical example of 1D conductive material^13^, whose band gap can be tuned as a function of the nanoribbon width and edge structure. Therefore, it has demonstrated exceptional electronic properties and several potential applications in electronic devices^14^. 1D quantum confinement and structural anisotropy can lead to interesting physical phenomena as well as applications in flexible electronic devices^15,16^. Recently, owing to their high structural regularity, chemically modular edges, and tunable structure–activity relationship, the construction of nanomaterials based on small molecular building blocks has garnered significant attention^17^. Although there are many reports on the synthesis of linear, planar, and stereoscopic polymers with diverse structures and properties, the synthesis of crystalline 1D organic nanomaterials, which includes repetition of highly conjugated structural units with stable ordered structure and excellent conductivity, has met with much less success. Therefore, the precise construction of 1D conductive nanomaterials through electronically conjugated metal-organic frameworks (MOFs) or covalent organic frameworks (COFs) is of immense significance to boost the applications of such materials.

**Fig. 1:**
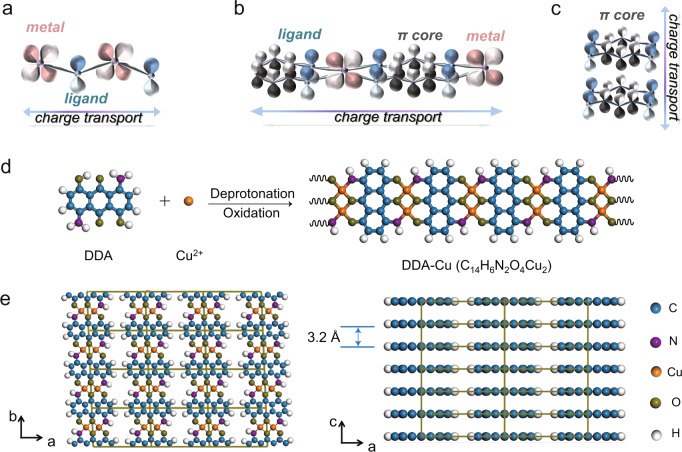
Charge transport pathways of MOFs and synthesis process of DDA-Cu MOF. **a–c** Charge transport pathways through-bond (**a**), extended conjugation planes (**b**) and through-space (**c**). The “metal” means the *d* orbitals of metals, “ligand” is the *p* orbitals of ligand and “π core” represents the conjugated *π* plane of ligand. **d** Synthetic schematic diagram of DDA-Cu. **e** Crystal structure of DDA-Cu.

Building effective charge transport pathways, such as through-bond pathways, extended conjugation pathways, and through-space pathways, plays a crucial role in the preparation of conductive MOFs^18^. For through-bond pathways (Fig. 1a), metal ions with different coordination numbers dominate the coordination bond paths of MOFs. Particularly, active *d*-electron transition metals, which have a suitable radius to obtain a better orbital overlap with the ligands, are conducive to generating a small band gap and high charge mobility. By utilizing ligands with aromatic organic cores, coordination bonds can evolve into extended *π–d-*conjugated planes (Fig. 1b), while *π–π* stacking of aromatic organic cores in the ligands causes the through-space pathways to be perpendicular to the conjugated planes in 2D materials (Fig. 1c). Especially, in some lanthanide MOFs^19,20^ and 2D COFs^21–23^, the significant contribution of *π–π* stacking to the conductivity has been confirmed. By constructing *π–d*-conjugated planes and *π–π* stacking paths, 2D MOFs can occupy the dominant position in conductive MOFs^24–26^. Based on the strong in-plane conjugation between the *π-*systems of the ligands and the *d* orbitals of the metals, the highest reported conductivity values of non-porous crystalline copper (II) benzenehexathiolate coordination polymers^27^ and porous Ni-based MOFs^25^ are 2500 and 40 S cm^−1^, respectively.

Theoretically, 1D MOFs have a higher structural degree of freedom than 2D and 3D MOFs, providing more opportunities for creating conductive paths. However, for 1D MOFs, the chain stacking is adjusted by van der Waals or hydrogen bond interactions with only one dimension restricted by the coordination bonds. This creates several challenges in designing the spatial arrangement of 1D chains to improve conductivity. Here, focusing on the construction of effective charge transport pathways, we have designed and synthesized a 1D DDA-Cu MOF with nanoribbon layers based on the self-assembly of Cu^2+^ and DDA ligand with an extended *π* plane of the anthraquinone nucleus (Fig. 1d). Surprisingly, in the plane, the DDA-Cu chains, called nanoribbons, form extended *π–d*-conjugated planes with the configuration restrictions of the bimetallic Cu^2+^ sites, and the chains arrange in a nanoribbon layer by van der Waals interaction similar to the 2D MOF layer. In addition, the DDA-Cu exhibits a tight *π–π* stacking outside the plane. As expected, the DDA-Cu exhibits excellent conductivity of 9.4 S m^−1^, one of the highest reported values for 1D conductive MOFs. Density functional theory (DFT) calculations are utilized to confirm that the constructed out-of-plane charge transport pathways are responsible for excellent conductivity. Further, the good electrical performance of the DDA-Cu is validated by employing it as an active material for symmetrical supercapacitors. The mass capacitance and areal capacitance reach 118 F/g and 236 mF/cm^2^, respectively, exceeding the values of many recently reported carbon materials (carbon nanotubes, graphene, MOFs, etc.). In addition, a flexible photoelectric synaptic device based on DDA-Cu films is also manufactured, and it effectively mimics biological synapse-like signals, including postsynaptic current, paired-pulse facilitation (PPF), short-term plasticity (STP), long-term plasticity (LTP), and STP-to-LTP transformation. Interestingly, the flexible device array with good air stability and bending resistance achieves an imaging function similar to human visual perception.

## Results

### Synthesis and characterization

Figure 1d displays a schematic of the synthetic route of DDA-Cu and the detailed reaction mechanism is presented in Supplementary Fig. 1. We selected hexacoordinated DDA as the organic building block for the coordination reaction with Cu^2+^ ions and obtained crystalline DDA-Cu MOF with isolated yields of 85–92% in a solution of ethanol and water. Due to the high restriction of bimetallic sites on the structure planarity, the DDA-Cu grows linearly along the coordination nodes, and the 1D nanoribbons stack through non-bonding interactions to form bulk materials (Fig. 1e).

**Fig. 2:**
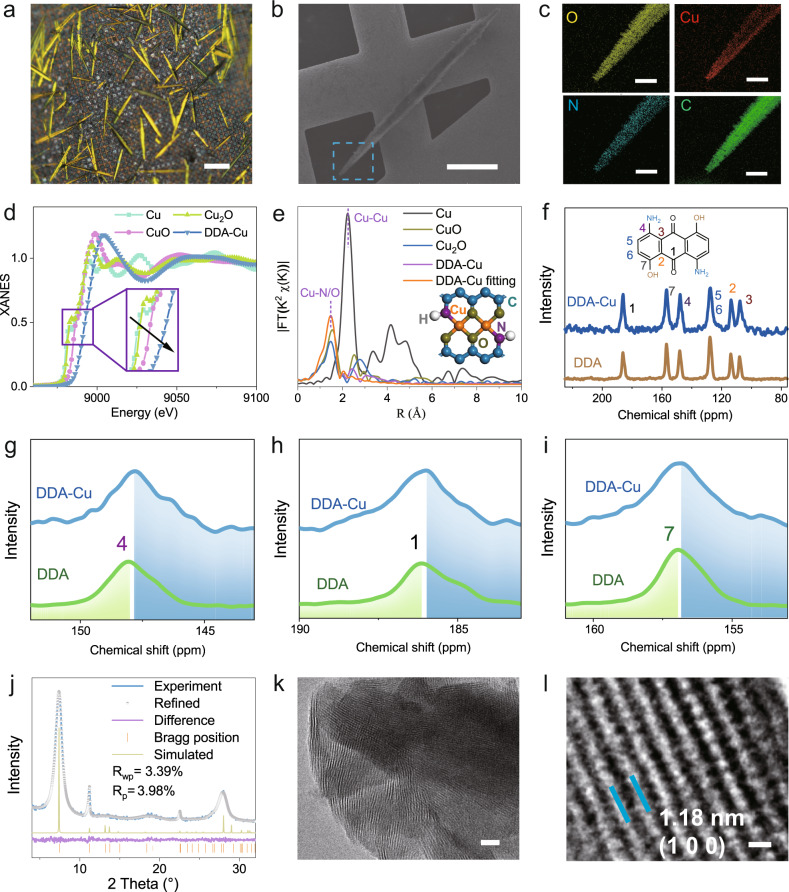
Morphology and structure characterizations of DDA-Cu. **a** Optical image of DDA-Cu crystals. Scale bar 25 μm. **b** SEM image of a DDA-Cu crystal. The scale bar is 25 μm. **c** Element mapping of the DDA-Cu crystal. Scale bar 5 μm. **d** XANES profiles of DDA-Cu, CuO, Cu_2_O, and Cu. **e** EXAFS curves of DDA-Cu, Cu_2_O, CuO and Cu. **f** ^13^C CP/MAS NMR spectra for DDA-Cu and DDA. **g**–**i** Partial enlarged view of carbon atoms 4, 1 and 7 in DDA-Cu and DDA. **j** PXRD results of DDA–Cu. **k**, **l** TEM images of DDA–Cu with different magnifications. Scale bars 10 nm in (**k**) and 1 nm in (**l**). Source data are provided as a Source Data file.

As shown in Fig. 2a and b, the DDA-Cu MOF has a needle-like crystalline morphology with a maximum length of more than 100 μm. The elemental mapping (Fig. 2c) demonstrates a homogeneous distribution of O, Cu, N and C at the end of the crystal. In the X-ray photoelectron spectra (XPS) (see Supplementary Fig. 2a), the two main peaks at 953.8 and 933.9 eV are ascribed to Cu 2*p*_1/2_ and Cu 2*p*_3/2_, respectively, which are consistent with the values reported for Cu-based frameworks^28^. The electron paramagnetic resonance (EPR) spectrum of DDA-Cu displays a clear signal (*g* = 2.12), which is attributed to the single electron of 3*d*^9^ orbital of Cu^2+^ (see Supplementary Fig. 3). The line intensity for DDA-Cu in the X-ray absorption near edge spectrum (XANES; Fig. 2d) is closer to that for CuO, indicating higher energy than Cu_2_O and Cu, which demonstrates the formation of Cu^2+^ species in the MOF. The extended X-ray absorption fine structure (EXAFS) reveals the presence of metal coordination structure in the DDA-Cu (Fig. 2e). The EXAFS fitting curves show that the coordination number of the Cu site is 4 with the distance of 1.82 Å for Cu–O and 1.96 Å for Cu–N. Furthermore, the maximum intensity at ~5 Å^–1^ in the wavelet transform (WT) of the EXAFS indicates the Cu–O/N contribution (see Supplementary Fig. 4).

The solid-state ^13^C cross-polarization/magic angle spinning nuclear magnetic resonance (^13^C CP/MAS NMR) spectra demonstrate the organic structure of DDA-Cu. As shown in Fig. 2f, the organic C skeleton of DDA-Cu is consistent with that of the DDA ligand, and the chemical shifts of C connected to the amino group (4), carbonyl group (1), and hydroxyl group (7) have a significant change (Fig. 2g–i), causing the transfer of 2*p* electrons in N/O to the 3*d* orbital of Cu^2+^. This is also supported by the shift of N/O toward higher binding energy in XPS of DDA-Cu (see Supplementary Fig. 2). The elemental analysis by inductively coupled plasma-optical emission spectrometry (ICP-OES) suggests that the content of Cu in DDA-Cu is 30.05%, which is consistent with the theoretical value of 32.5%.

Figure 2j shows the powder X-ray diffraction (PXRD) pattern of the crystalline DDA-Cu. The PXRD pattern indicates that DDA-Cu is a crystalline material with a long-range structure. The experimental (blue curve) and theoretical (grey curve) results are in good agreement with each other. The Pawley refinement result (*R*_p_ = 2.5%, and *R*_wp_ = 3.5%) proves that the cell parameters of DDA-Cu are *a* = 11.8 Å, *b* = 7.9 Å, *c* = 3.2 Å, *α* = *β* = 90.0°, and *γ* = 87.7° in the P2/M space group (the crystal structure is presented in Table S1). Five diffraction peaks at 2*θ* = 7.4°, 11.2°, 18.4°, 22.5°, 127.9° are observed, which can be indexed as the (1 0 0), (0 1 0), (2 1 0), (0 2 0), and (0 0 1) reflections. The presence of a high-intensity peak at 7.4° is associated with the periodicities in the (1 0 0) plane and indicates a well-defined ordered columnar array. In addition, for comparison, aligned, dislocated and rotation structures are also predicted in which one Cu^2+^ ion is coordinated with two organic ligands (see Supplementary Figs. 5 and 6). For the dislocated one (Model A), two small peaks are located at 9.2° and 9.5°. For the aligned one (Model B), there is no peak in the range from 8° to 12°. These features are not observed in our samples, indicating the sole formation of crystalline DDA-Cu with bimetallic Cu^2+^ coordination at the joint point (Fig. 1e).

The N_2_/Ar adsorption/desorption isotherms of DDA-Cu are shown in Supplementary Fig. 7. The calculated Brunauer–Emmett–Teller surface area is ~127.3 m^2^/g (N_2_). The high-resolution transmission electron microscopy (HRTEM) image demonstrates the 1D periodicity of DDA-Cu, and the lattice fringe spacing is ~1.18 nm, which corresponds to the (1 0 0) plane (Fig. 2k and l). Further, the lattice fringe spacing of ~0.32 nm corresponding to the (0 0 1) plane demonstrates its layered structure, which is similar to the 2D graphene structure (see Supplementary Fig. 8). The chemical stability of DDA-Cu was evaluated in Supplementary Fig. 9. In addition, thermal gravimetric analysis reveals an initial decomposition temperature of approximately 320 °C (see Supplementary Fig. 10), suggesting good thermal stability of the DDA-Cu.

**Fig. 3:**
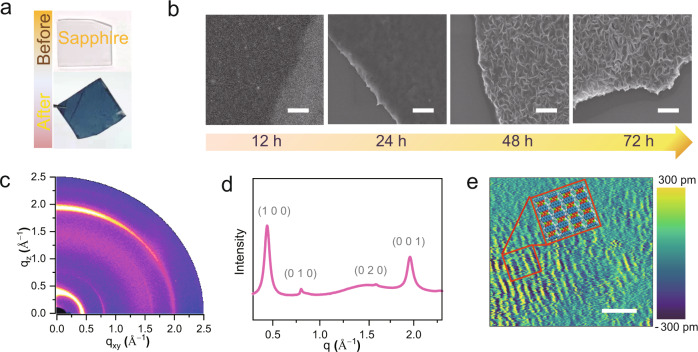
Morphology and crystallinity characterizations of DDA-Cu films. **a** Optical image of DDA-Cu film transferred on sapphire. **b** SEM images of DDA-Cu films grown at different reaction times. Scale bar 1 μm. **c** 2D GIWAXS image of a DDA-Cu film. Scale bar 4 nm. **d** The integral curve of GIWAXS result. **e** HRAFM of DDA-Cu film. Scale bar 4 nm. Source data are provided as a Source Data file.

Similar to the 2D *π*-conjugated COFs and MOFs systems, the 1D DDA-Cu displays strong charge delocalization across the entire nanoribbon plane, which is caused by the good energy overlap between the bimetallic nodes and the organic ligands in oxidized form. This property, along with the layered structure, makes the 1D DDA-Cu an ideal candidate to produce high-quality films by a liquid/liquid interface method that can preserve the electronic properties of the ribbons. DDA-Cu films can be transferred on sapphire and Si/SiO_2_ substrates (see Fig. 3a and Supplementary Fig. 11), and their thickness can be controlled by the growth time (see Supplementary Fig. 12). The film is ~8 nm thick after 12 h growth, and after a longer growth time of 72 h, the thickness can reach nearly 245 nm. Further, its morphology changes from an early dense film to a loose leaf stacked on the surface (Fig. 3b). For a 25-nm-thick film, the 2D grazing-incidence wide-angle X-ray scattering (GIWAXS) image (Fig. 3c) and the integral curve (Fig. 3d) prove the crystallinity of the film, and the four diffraction signals at *q* = 0.44, 0.81, 1.6, and 1.9 Å^–1^ are identified, which are in accordance with the PXRD results. According to the intensity variation of the (0 0 1) diffraction ring (1.9 Å^–1^) in the in-plane and out-of-plane direction of GIWAXS profiles, a preferentially oriented film is formed on the substrate surface (see Supplementary Fig. 13). The structure of DDA-Cu film is also investigated by high-resolution atomic force microscopy (HRAFM; see Fig. 3e and Supplementary Fig. 14), where parallel patterns with a width of ~1.2 nm are observed. In addition, HRTEM images show that the film consists of some highly ordered, perfectly aligned small domains that correspond to the (1 0 0) plane (see Supplementary Fig. 15), indicating the high-crystalline quality of the DDA-Cu film.

### Electrical properties

The conductivity of the DDA-Cu film transferred on a Si/SiO_2_ substrate was measured through two-terminal devices (see Supplementary Fig. 16), and the results are shown in Supplementary Fig. 17. The linear and symmetric current–voltage (*I*–*V*) curves suggest that ohmic contacts are formed at the source and drain electrodes. The obtained conductivity is ~9.4 S m^−1^, which is at least two orders of magnitude higher than that of conventional 1D MOFs^29,30^ (Table S2) and is comparable to that of some conductive MOF and COF films^23,28^ (Table S3). The impressive conductivity makes it a promising candidate for energy storage applications. To this end, we studied its capacitance performance through a three-electrode system in a 3 M KOH aqueous solution. The cyclic voltammetry (CV) curves have no typical redox waves under both cathodic (−0.4 to 0.0 V) and anodic (0.0–0.6 V) cycling (see Supplementary Fig. 18a), suggesting a double-layer capacitance behavior^31^ in a wide voltage window of 1 V. The charge storage mechanism is discussed in detail in Supplementary Figs. 19 and 20. Subsequently, we fabricated a symmetrical supercapacitor with DDA-Cu as the active electrode material. The CV curves exhibit good reversibility at different scan rates (see Supplementary Fig. 18b) similar to the curves of 2D MOF electrode^32^, demonstrating a stable charge storage behavior of this device. The calculated specific gravimetric capacitance (*C*_g_) using the galvanostatic charge–discharge (GCD) curves at different current densities is shown in Supplementary Fig. 18c. At a low charge–discharge rate of 0.1 A/g, the *C*_g_ is 118 F/g, which is superior to that of many reported activated carbons, carbon nanotubes, graphene, and MOFs (Table S4). Furthermore, under a current density of 0.4 mA/cm^2^, the calculated maximum areal capacitance (*C*_s_) is 236 mF/cm^2^, which is significantly better than the reported values for some MOF-based supercapacitors^33–36^. The ion and charge transport kinetics of the device was examined by electrochemical impedance spectroscopy (EIS). As displayed in the Nyquist plots (see Supplementary Fig. 18d), the small semicircle at a high frequency indicates low charge transfer resistance (*R*_ct_, 1.3 Ω), and low equivalent resistance (*R*_s_, 3.2 Ω), demonstrating efficient charge transfer and electrolyte ion transport^24,32^.

### Band structure

**Fig. 4:**
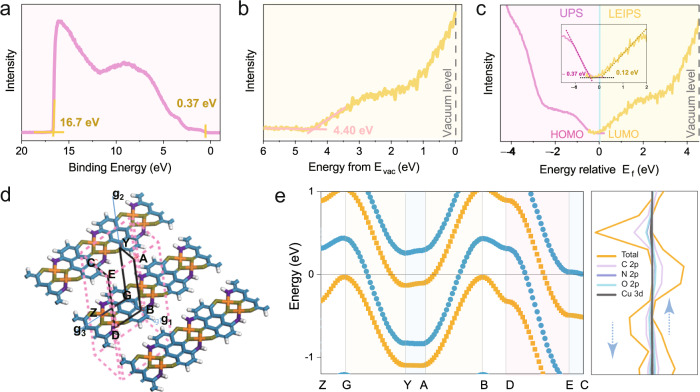
The band structure characterizations of DDA-Cu film. **a** The UPS spectroscopy of DDA-Cu. **b** The LEIPS spectroscopy of DDA-Cu. **c** The electrical band gap characterization combined by UPS and LEIPS. **d** A view of the Brillouin zone paths used in the DFT calculations. **e** The band structure (the left part) and density of states (the right part) for DDA-Cu by HSE06 method. The Fermi energy has been corrected to 0 eV. The orange and blue branches correspond to spin-up and spin-down bands in the band structure, respectively. For the density of states, the arrow up represents the spin-up part and the arrow down represents the spin-down part. Source data are provided as a Source Data file.

Characterizing the band structure through experimental and theoretical methods can be useful to comprehensively explore the electronic structure of the DDA-Cu and understand its intrinsic conduction mechanism. Figure 4a shows the ultraviolet photoelectron spectrum (UPS) of DDA-Cu, where the highest occupied molecular orbital (HOMO) is 0.37 eV below the Fermi level (*E*_f_). Traditionally, the lowest unoccupied molecular orbital (LUMO) of organic materials is estimated based on the optical band gap (*E*_g_^opt^) because it is challenging to determine a reliable LUMO value through experiments^37^. However, via low-energy inverse photoelectron spectroscopy (LEIPS) with low damage to film materials, we show an example that the experimental LUMO of MOFs can be measured directly, which is 4.40 eV below the vacuum level (*E*_vac_) for DDA-Cu (Fig. 4b). Therefore, the electrical band gap (*E*_g_) of DDA-Cu is calculated to be 0.49 eV, where the *E*_f_ is set as 0 (Fig. 4c). This is one of the typical narrow band gaps reported for MOFs^19,20,38^, which facilitates easy charge transportation. And we performed variable temperature conductance tests (see Supplementary Fig. 21), Hall effect tests (see Supplementary Fig. 22a) by van der Pauw technique, and Motto–Schotky curve test (see Supplementary Fig. 22b) proving the n-type semiconducting properties of DDA-Cu. In addition, the *E*_g_^opt^ of DDA-Cu is estimated through infrared diffuse reflection spectra (see Supplementary Fig. 23), and the indirect *E*_g_^opt^ is obtained to be 0.19 eV. Then, the exciton binding energy (*E*_b_ = *E*_g_–*E*_g_^opt^) is ~0.3 eV (see Supplementary Fig. 24), which represents the energy binding an electron–hole pair through the electrostatic Coulomb force in DDA-Cu^39,40^. The *E*_b_ is close to the minimum value achieved for 2D metal halide perovskites and transition-metal dichalcogenides (TMDCs)^41,42^, which facilitates high-efficiency charge separation in DDA-Cu with potential application in optoelectronic devices.

Further, DFT calculations based on HSE06 method were utilized to examine the band structure of the DDA-Cu. The Brillouin zone paths are shown in Fig. 4d. The band structure and projected density of states (PDOS) of DDA-Cu are shown in Fig. 4e. Specifically, in the band structure, along the direction of interlayer *π–π* stacking (G–Y, A–B, and D–E), the band crosses the Fermi level, which suggests metallic characteristics of the MOF as the optimal charge transport path. By contrast, the Fermi level is located inside the bands along the in-plane direction (Z–G, Y–A, B–D and E–C), indicating a semiconductor behavior. An in-plane indirect band gap of 0.34 eV at the G and Z points (see Supplementary Fig. 25) was observed. In fact, in the in-plane direction, the band also presents significant anisotropy: significant dispersion occurs along the chain direction (Z–G and B–D) in the band; however, the band is almost flat in the direction perpendicular to the DDA-Cu chain (Y–A and E–C) as the most unfavorable charge transport path. These results indicate that DDA-Cu is rich in anisotropic transport behavior^19^.

### Flexible organic optoelectronic synapse

**Fig. 5:**
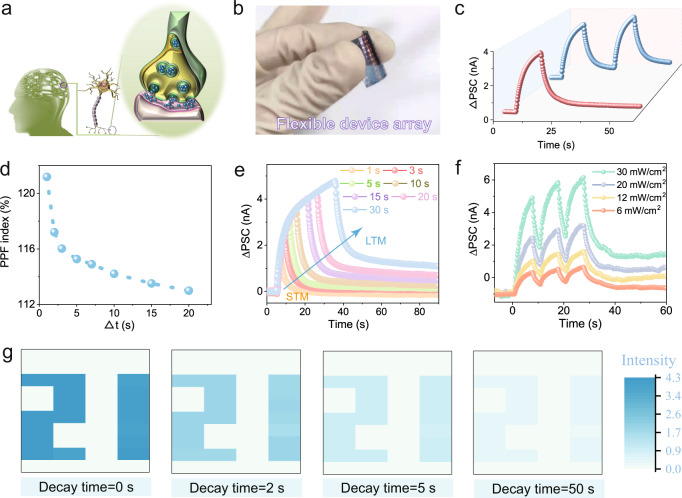
Flexible organic optoelectronic synapse. **a** Schematic diagram of synapses in the human brain. **b** The optical image of a DDA-Cu-based flexible device on PET substrate. **c** Synaptic signals of the flexible devices based on PET substrate stimulated by one light pulse (red) and two light pulses (blue) under white light (excitation time = 10 s, interval time = 15 s between two light pulses, 20 mW/cm^2^). The *V*_DS_ is 5 V. **d** Variation curve of PPF with △*t*. **e** The transition from STP to LTP by the duration of different light stimuli. **f** The transition of STP to LTP is introduced by different light intensities. **g** Diagram of the image memory of the number “21”. Source data are provided as a Source Data file.

Narrow *E*_g_ and small *E*_b_ are conducive to achieving charge excitation and efficient charge separation^39^. Thus, based on the DDA-Cu films coated on Si/SiO_2_, we fabricated optoelectronic devices and explored their potential as an active semiconductor layer in the simulation of human brain synapses (Fig. 5a). The current variation caused by light excitation can simulate postsynaptic current change (∆PSC) during the nerve signal transmission process of the human brain. The proposed photo-synaptic mechanism is presented in Supplementary Fig. 26 and we also analyzed the influence of the thermal effect of lighting (see Supplementary Fig. 27). We investigated the ∆PSC characteristics under different excitation wavelengths in the device. The ∆PSC of red light is higher than that of yellow light (see Supplementary Fig. 28), which is consistent with the ultraviolet–visible–near-infrared (UV–vis–NIR) absorption spectrum (see Supplementary Fig. 29), and white light corresponds to the most significant excitation current. Therefore, white light was used to investigate the performance of the device. As shown in Supplementary Fig. 30a, since the photogenerated electron–hole pairs in the DDA-Cu cannot recombine immediately after the light is removed^43^, the current decreases slowly (∆PSC = 5.0 nA) after the light source is removed. Further, ∆PSC increases to a higher value of 5.5 nA after two light pulses (see Supplementary Fig. 30b).

The ever-increasing demand for flexible electronics around the world prompted us to evaluate the application of DDA-Cu film in flexible devices (Fig. 5b). Compared with the devices using the Si/SiO_2_ as substrate, the synaptic response has only a slight decrease (∆PSC = 3.6 nA by one light pulse and ∆PSC = 4.1 nA by two light pulses in Fig. 5c and Supplementary Fig. 30c,d) in the devices based on polyethylene terephthalate (PET). The reproducibility of the photo-synaptic behavior described above is shown in Supplementary Figs. 31 and 32. PPF is an important manifestation of synaptic STP, which is defined as the ratio of the second ∆PSC to the first ∆PSC after two light pulses. Figure 5d shows the variation in the PPF as a function of the time interval (∆*t*). As the ∆*t* increases, the PPF value gradually decreases from 121% to 112%, and this is similar to the positive correlation between memory strength and memory interval in the human brain learning process, which is essential for recognizing and decoding time-resolved information (Table S5)^44–46^. Repeated rehearsal of STP gradually leads to LTP. The transformable characteristics of synaptic plasticity enable synapses to provide specific responses to complex external stimuli. When the light lasts for 1 s, the ∆PSC rapidly decreases to 0 after the light is removed (Fig. 5e). However, when the illumination time increases to 30 s, the ∆PSC increases significantly (∆PSC = 4.8 nA). After the light is turned off, the current remains at a high value and it takes more time to decay to 0, which demonstrates the typical transition from STP to LTP by radiation time. We also fitted the decay curves of different irradiation times and studied the effect of the duration time of light on the time constant (see Supplementary Fig. 33). Meanwhile, after the light is turned off, the device exhibits higher current as the frequency gradually increases from 0.02 to 0.25 Hz (see Supplementary Fig. 34), which proves that the radiation frequency can also effectively realize the transition from STP to LTP. The light spike number can also accomplish the device transition STP to LTP (see Supplementary Fig. 35). When the spike number is increased from 3 to 16, ∆PSC reaches a larger value, and a higher steady-state current is obtained after the light is turned off. Similarly, the light intensity can also trigger this transition (Fig. 5f). These pieces of evidence of the transition from STP to LTP imply that the devices are beneficial to simulate the human-like short-term memory and long-term memory behaviors for application in smart microelectronic devices.

Furthermore, we integrated a 4 × 10 photo synapse array to simulate the human-like visual perception function, and the image recognition and memory functions of the devices are evaluated by irradiating the patterned light (see Supplementary Fig. 36). The number pattern “21” is successfully resolved after the light is turned on (Fig. 5g), demonstrating the reliable imaging function of the device. Additionally, after the light source is removed, the “21” pattern is memorized and remains clearly visible until 50 s later, verifying that the memory and memory loss behaviors are successfully imitated. It may be noted that the ∆PSC distribution of the imaging point under and after the illumination is narrow with small fluctuations, proving the high stability and uniformity of the human-like visual perception system. Specifically, after being exposed to air for 45 days, the ∆PSC of the device shows a minor decrease (see Supplementary Fig. 37), and after 500 bending experiments (see Supplementary Fig. 38), ∆PSC still remains at 70% (see Supplementary Fig. 39), indicating the excellent bending resistance and air stability. The results suggest that the flexible DDA-Cu-based devices can effectively simulate the artificial vision system with excellent stability.

## Discussion

We fabricated a conductive 1D DDA-Cu MOF with extended *π–d*-conjugated nanoribbon layers. The experimental results showed that the DDA-Cu is a typical narrow band gap semiconductor (0.49 eV) with a small *E*_b_ of 0.3 eV. It exhibited an impressive conductivity of ~9.4 S m^−1^, which was attributed to the efficient charge transport along the DDA-Cu chains and the *π–π* stacking direction, as confirmed by DFT calculations. When used as the active material for a supercapacitor, the DDA-Cu exhibited excellent charge storage capacity and stability. Furthermore, as the active layer of photosynaptic device, it successfully simulated the artificial visual perception system with an imaging function, excellent bending resistance, and air stability, which has immense potential in human-like visual simulation.

## Methods

### Materials

Cu(CH_3_COO)_2_·H_2_O, 1,5-Diamino-4,8-dihydroxyanthraquinone (DDA) were purchased from SAAN Chemical Technology Co. Ltd. Ethanol, and tetrahydrofuran and dichloromethane (CH_2_Cl_2_) were purchased from Concord Technology Co., Ltd. All reagents were utilized without purification.

### Synthesis of DDA-Cu MOF crystals

30 mg of DDA was added 400 mL ethanol and 100 mL of water (solution A). 22.2 mg Cu(CH_3_COO)_2_·H_2_O was dissolved in a mixed solution of 400 mL ethanol and 100 mL water (solution B). Solution A was sonicated for 30 min to ensure dissolution. Solution A was stirred while adding solution B into solution A (about 2 drops per second). Then the mixed solution was stirred at 90 °C under normal pressure for 24 h. After centrifugation at 9000 rpm, the blue-black precipitate was collected and centrifuged repeatedly with ethanol and water until the supernatant was clear. The blue-black product was dried in a vacuum drying oven at 90 °C for 12 h.

### Synthesis of DDA-Cu MOF film

A liquid–liquid interface is manufactured for the interface coordination reaction of Cu^2+^ and DDA. The bottom layer is 200 mL CH_2_Cl_2_ containing 5.5 mg DDA and the top layer is 100 mL H_2_O solution containing 13.5 mg Cu(CH_3_COO)_2_·H_2_O. After standing for 3 days, a stable large-area DDA-Cu MOF film can be obtained at the interface.

### Characterization of DDA-Cu MOF

^13^C CP/MAS NMR spectroscopy was recorded on Bruker AVANCE III spectrometer (300 MHz). All chemical shifts are reported in parts per million (ppm). PXRD data were recorded on a powder x-ray diffractometer (Empyrean). TEM observations were conducted on a Cryo-transmission electron microscope (Themis 300). XPS measurements were finished with the ESCALAB250Xl spectrometer using Al Kα X-rays. The UV−vis−NIR absorption spectrum was taken on an ultraviolet, visible spectrophotometer (SHIMADZU, UV-2600). SEM images and element mapping were performed on the Hitachi S-4800 microscope. Thermogravimetric analysis was conducted in a nitrogen atmosphere with a heating rate of 5 ^o^C/min on a Pyris 1 TGA. The GIWAXS measurements were conducted at the synchrotron XRD facility at beamline No. 14B/15U at Shanghai Synchrotron Radiation Facility (SSRF). Elemental analysis of Cu in DDA-Cu MOF was tested by an Agilent 5110 inductively coupled plasma-optical emission spectrometer (ICP-OES). For XAS measurements, the DDA-Cu were analyzed by Cu K-edge XANES at the XAS beamline of the Australian Synchrotron. Energy in spectra has been calibrated, averaged and normalized utilizing the Athena software package^47^. EPR spectra were collected on a Bruker E500 electron paramagnetic resonance spectrometer at room temperature. N_2_ adsorption isotherm curves are obtained from the micromeritics ASAP 2460 3.01. Atomic force microscopy tests were conducted using a NanoMan VS AFM under the tapping mode. HRAFM images were obtained using CyPher ES AFM.

### Computation method

All the above calculations are conducted using the density functional theory with the projector-augmented-wave (PAW) method, as adopted in the Vienna ab initio simulation package (VASP). For the initial structure optimization of DDA-Cu, the generalized gradient approximation raised by Perdew, Burke, and Ernzerhof (GGA-PBE) is applied for the exchange-correlation potential. For an exact depiction of the localized *d* electrons of DDA-Cu, the on-site Coulomb interaction was considered to the *d* orbitals of Cu employing an appropriate *U* value (4.0 eV)^48^. The energy convergence criterion was 1 × 10^–6^ eV and the Hellmann Feynman force convergence criterion was 0.03 eV/Å. The kinetic energy cut-off of 500 eV was applied. Subsequently, via the HSE06 method, a hybrid functional with 1/4 exact Harteen–Fock exchange term, more accurate structural relaxation of DDA-Cu and band structure calculation with the equilibrium geometry of DDA-Cu were applied. It needs to be clarified that the calculation results are based on the perfect crystal model. When complex situations such as crystal defects (vacancy, distortion, etc.)/doping (caused by impurities, etc.)/possible oxidation forms must be considered, the applicability of the above results should be reassessed.

### Electrochemical measurement

To prepare the electrode, DDA-Cu crystals, polytetrafluoroethylene, and conductive carbon black with a mass ratio of 7:1:2 were mixed and ground into jelly and coated on 1 × 1 cm nickel foams. The electrode is dried at 90 °C for 8 h and fully dried, and the mass of the active materials is about 4 mg for one single electrode using all measurements. The prepared DDA-Cu on nickel foams as the working electrode is used for a three-electrode test in a 3 M KOH aqueous solution with a Pt plate as the counter electrode and Ag/AgCl as the reference electrode using a CHI 760E electrochemical equipment. Two pieces of DDA-Cu electrodes are separated by a diaphragm impregnated with 3 M KOH to prepare the symmetrical supercapacitors and wrapped plastic wrap on the outside to reduce electrolyte volatilization. According to the GCD curves, the specific capacitance is calculated according to the following formulas:

1 $${C}_{{\rm {g}}}=4\times \left(\frac{I}{m}\right)\times \frac{\triangle t}{\triangle V},$$

2 $${C}_{{\rm {s}}}=2\times \left(\frac{I}{S}\right)\times \frac{\triangle t}{\triangle V},$$

where $\frac{I}{m}$ (A/g) and $\frac{I}{S}$ (mA/cm^2^) are the current density,$\,\triangle t$ represents the discharge time,$\,\triangle V$ means the voltage window. For the electrochemical impedance spectroscopy (EIS) test, the frequency was changed from 10^5^ to 0.01 Hz at open circuit potential with an amplitude of 10 mV.

### Electronic device

DDA-Cu film was transferred on SiO_2_/Si substrates (300 nm SiO_2_ as a dielectric layer), which were coated with interdigital electrodes. The thickness of Au electrodes is 30 nm. The *I*–*V* curve measurements were conducted in an N_2_ atmosphere at room temperature. The conductivity (*k*, S m^–1^) was evaluated as

3 $$k=\frac{I}{E}\times \frac{l}{d\times h},$$

where $I$ is the channel current (A), $E$ is the source–drain voltage, $l$ (m) is the channel length, $d$ (m) is the channel width and $h$ (m) is the thickness of DDA-Cu film, respectively. The size of *l* and *d* used for devices is 50 and 1400 μm. Da-Cu films for *I*–V tests were obtained at 12 h (growth time) and the thickness is about 8 nm. The flexible organic optoelectronic synapse devices were built by transferring the film on PET substrates. Si/SiO_2_ and PET substrates have been cleaned by immersion in water and ethanol and dried with nitrogen before use. All device performance tests are done on Keithley 4200SC semiconductor parameter analyzer.

## Supplementary information

Supplementary Information

## Data Availability

We have declared that all data supporting the findings of this study are available within the paper and its Supplementary Information. Source data are provided with this paper.

## Source data

Source Data
