# Publisher Correction: A one-dimensional conductive metal-organic framework with extended *π*-*d* conjugated nanoribbon layers

**DOI:** 10.1038/s41467-023-35884-8

**Published:** 2023-01-12

**Authors:** Shengcong Shang, Changsheng Du, Youxing Liu, Minghui Liu, Xinyu Wang, Wenqiang Gao, Ye Zou, Jichen Dong, Yunqi Liu, Jianyi Chen

**Affiliations:** 1 Beijing National Laboratory for Molecular Sciences, Key Laboratory of Organic Solids, Institute of Chemistry, Chinese Academy of Sciences, 100190 Beijing, P. R. China; 2 University of Chinese Academy of Sciences, 100049 Beijing, P. R. China

**Keywords:** Ligands, Electronic properties and materials, Electronic devices

Correction to: *Nature Communications* 10.1038/s41467-022-35315-0, published online 09 December 2022

The original version of this Article incorrectly made the peer review reports for this paper available. In the correct HTML version, the link to the peer review file has been removed. The second sentence of the Peer Review Information section, which stated ‘Peer reviewer reports are available’ has been removed. This has been corrected in both the PDF and HTML versions of the Article.

