# Supplementary material for: A one-dimensional conductive metal-organic framework with extended π-d conjugated nanoribbon layers
Source: Nat Commun. 2022 Dec 9;13:7599. doi: 10.1038/s41467-022-35315-0 (PMC9734122; doi:10.1038/s41467-022-35315-0)
Supplement: Supplementary file 1 — Supplementary Information [file 41467_2022_35315_MOESM1_ESM.pdf]

Supplementary Information

A one-dimensional conductive metal-organic framework with extended π-d conjugated nanoribbon layers

Shang et al.

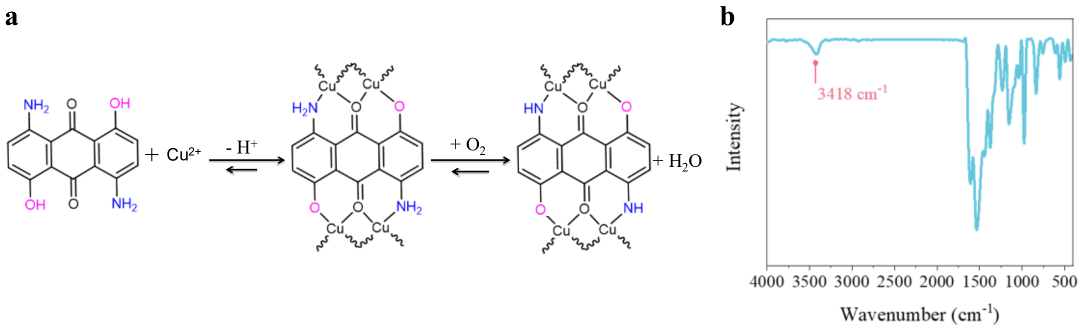

Supplementary Figure 1. Chemical structure and FTIR characterization of DDA-Cu. **a,** Schematic diagram of the preparation reaction mechanism. **b**, FTIR spectra of DDA-Cu.

Here the preparation reactions of the DDA-Cu involve the deprotonation of hydroxyl groups and the oxidation of amino groups. Hydroxyl protons go into solution by dissociation[1] and are subsequently captured by acetate without being converted to water molecules. Amino groups are oxidized by oxygen in the air to form water molecules, that is $4(-NH_2) + O_2 \rightarrow 4(-NH) + 2H_2O$, this process is the same as the synthesis of the two-dimensional amino ligand MOFs[2,3]. In Supplementary Fig. 1b, there is a –NH stretching vibration peak of 3418 cm^{-1} for DDA-Cu, which is consistent with the DDA-Cu structure described in the mechanism.

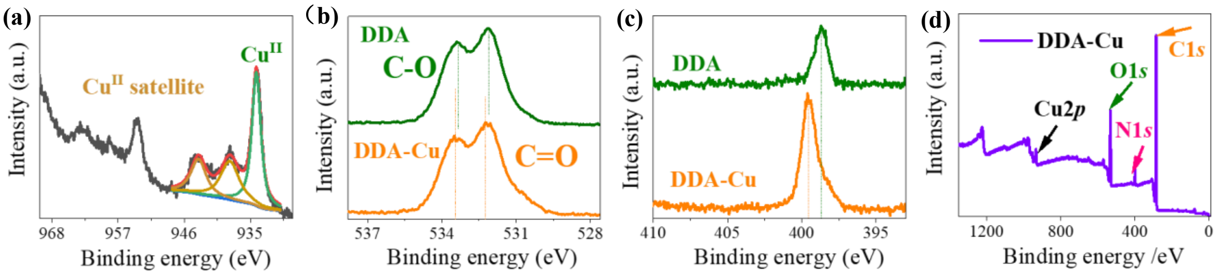

Supplementary Figure 2. XPS results of DDA and DDA-Cu. **a**, Cu 2*p* peaks of DDA-Cu. **b**, O 1*s* peaks of DDA and DDA-Cu. **c**, N 1*s* peaks of DDA and DDA-Cu. **d**, Full element curves for DDA-Cu.

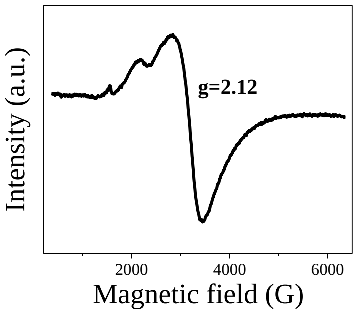

Supplementary Figure 3. EPR spectrum of DDA-Cu at the room temperature. The calculated g value is 2.2.

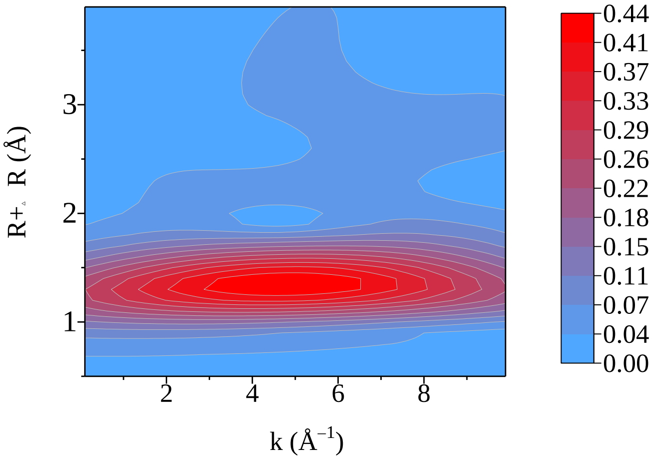

Supplementary Figure 4. Wavelet transform of the EXAFS.

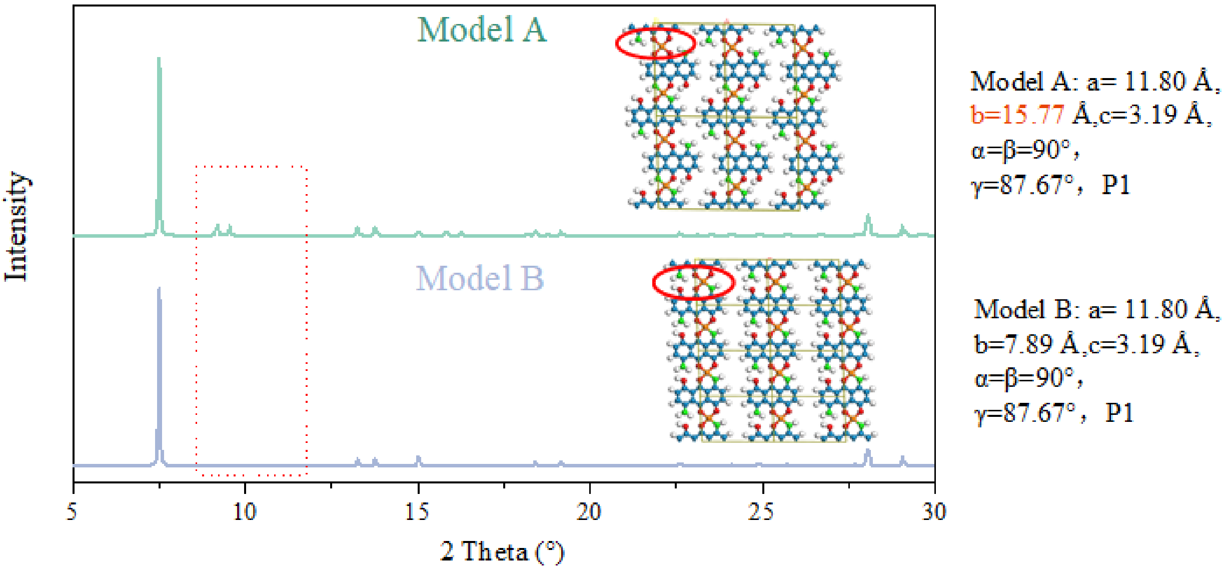

Supplementary Figure 5. Theoretical models and calculated XPD results of DDA-Cu with dislocated, and aligned structures. Model A: DDA-Cu with a dislocated structure. Model B: DDA-Cu with an aligned structure, and at each linkage point a Cu atom is removed.

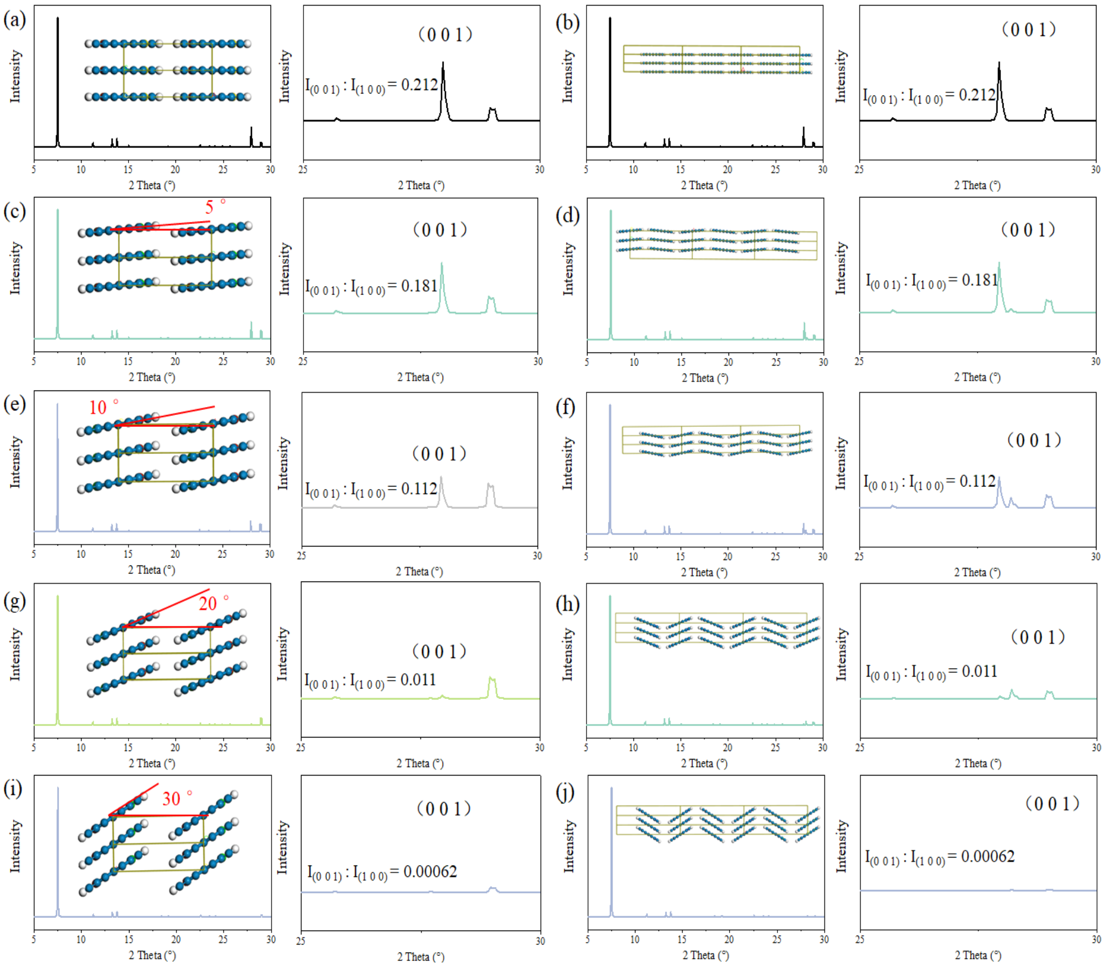

Supplementary Figure 6. Theoretical models and calculated XPD results of DDA-Cu with rotation structure in single cell and super cell. a–j, Rotation models with different rotation angles. Model a (rotation angle = 0°), c (rotation angle = 5°), e (rotation angle = 10°), g (rotation angle = 20°), i (rotation angle = 30°) take a single cell model with a = 11.8 Å, b = 7.9 Å, c = 3.2 Å, α = β = 90.0°, and γ = 87.7° in the P1 space group and all DDA-Cu chains rotate in the same direction. Model b (rotation angle = 0°), d (rotation angle = 5°), f (rotation angle = 10°), h (rotation angle = 20°), j (rotation angle = 30°) take a super cell model with a = 23.6 Å, b = 7.9 Å, c = 3.2 Å, α = β = 90.0°, and γ = 87.7° in the P1 space group and adjacent DDA-Cu chains rotate in the opposite direction.

With the increasing of rotation angles from 5° to 30° in single cell models and super cell models, the intensity peak of (001) planes decrease significantly due to the weakening of *π*-*π* layer stacking by rotation. Therefore, the $I_{(0\,0\,1)}$: $I_{(1\,0\,0)}$ decreases significantly from 0.212 to 0.00031. The $I_{(0\,0\,1)}$: $I_{(1\,0\,0)}$ value we observed experimentally is 0.27, which is the closest to the unrotated model (a and b), therefore the DDA-Cu are more inclined to align in the same plane and form a strong *π*-*π* stacking

between layers as evidenced by the strong (0 0 1) peak .

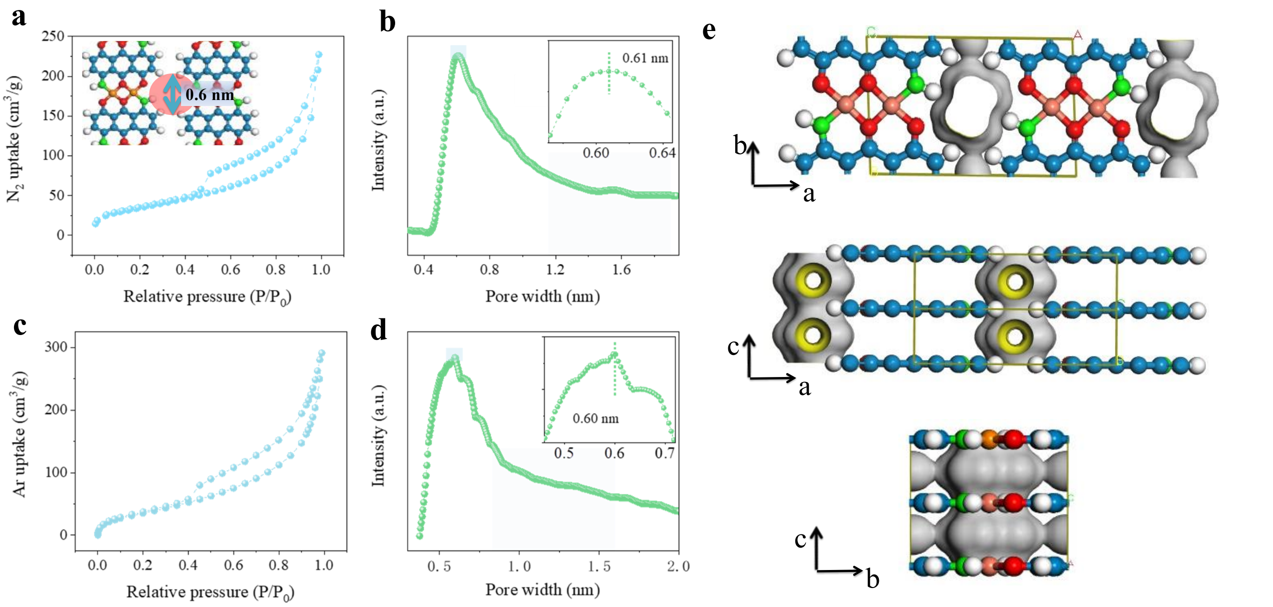

Supplementary Figure 7. Gas adsorption/desorption characterization. **a**,**b** N_2 adsorption/desorption isotherms and pore size distribution of DDA-Cu at 77 K. The BET surface areas are ~ 127.3 m^2/g (N_2) and 125 m^2/g (Ar). **c**,**d** Ar adsorption/desorption isotherms and pore size distribution of DDA-Cu at 87 K. **e**, Connolly surface analysis of DDA-Cu. Linear chains of DDA-Cu aligned in parallel form an inherent tubular pore structure.[4]

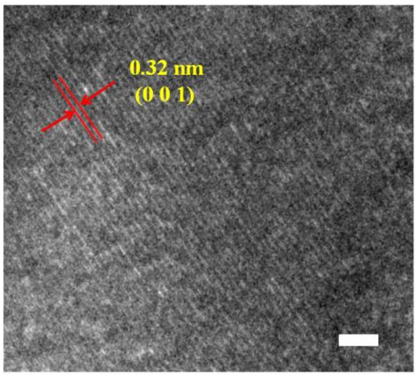

Supplementary Figure 8. HRTEM image of DDA-Cu. The HRTEM image showed that the DDA-Cu consist of parallel aligned 2D sheets, which indicates a highly ordered alignment. Scale bar 1 nm.

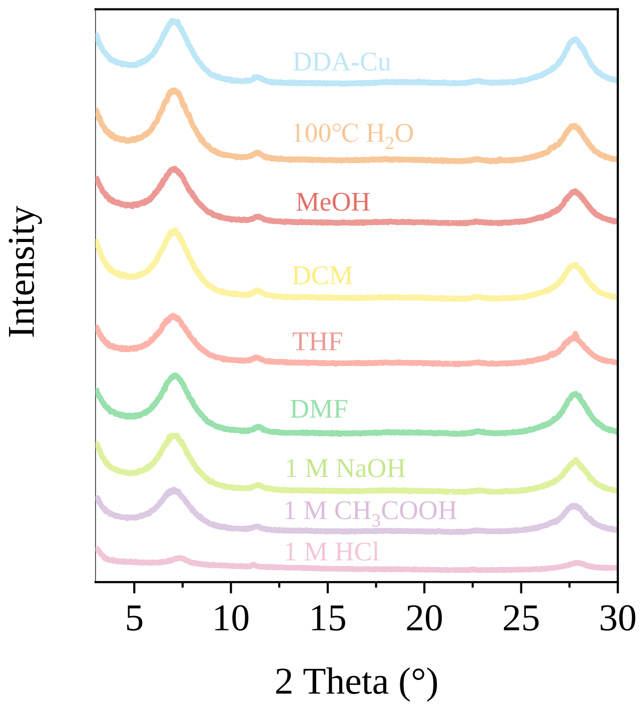

Supplementary Figure 9. Chemical stability of DDA-Cu. The soaking time was 24 hours in each experiment.

After being soaked in boiling water, MeOH, DCM, THF, DMF, and 1 M NaOH for 24 hours, the PXRD intensity only changed slightly, showing good solvent resistance, boiling water resistance, and alkali resistance. When immersed in 1 M CH_3COOH for 24 hours, the crystal structure of DDA-Cu was preserved to a certain extent. For 1M HCl, the crystallinity of DDA-Cu is almost completely lost. This is because the attack of proton (especially for strong acids, 1M HCl, etc.) on O/N atoms in DDA-Cu promotes the cleavage of the coordination bond, and then the MOF is decomposed.

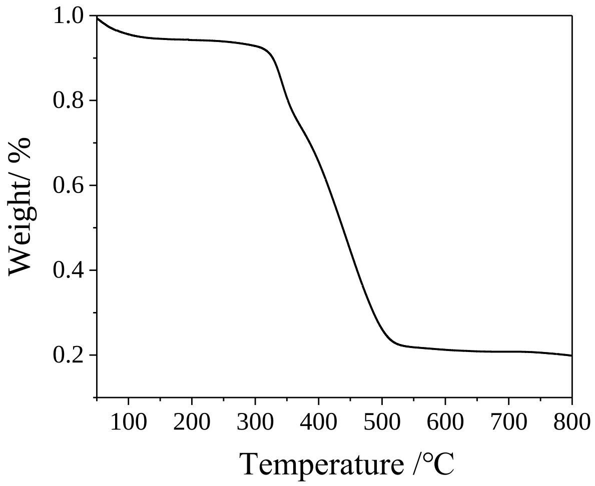

Supplementary Figure 10. TGA curve of DDA-Cu.

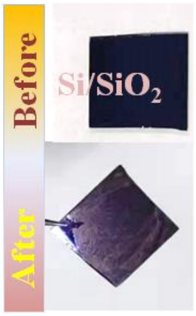

Supplementary Figure 11. DDA-Cu films transferred on Si/SiO$_2$ substrate.

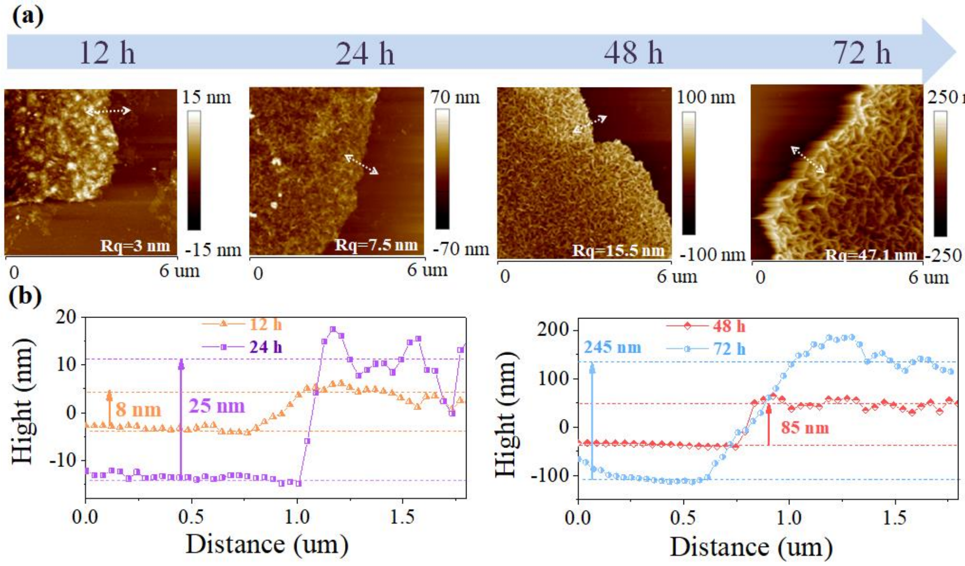

Supplementary Figure 12. AFM characterizations of DDA-Cu film. **a,** AFM images of DDA-Cu films grown at different time from 12h to 72 h. **b**, Corresponding height profiles across the smooth Si/SiO$_2$ substrate and DDA-Cu films.

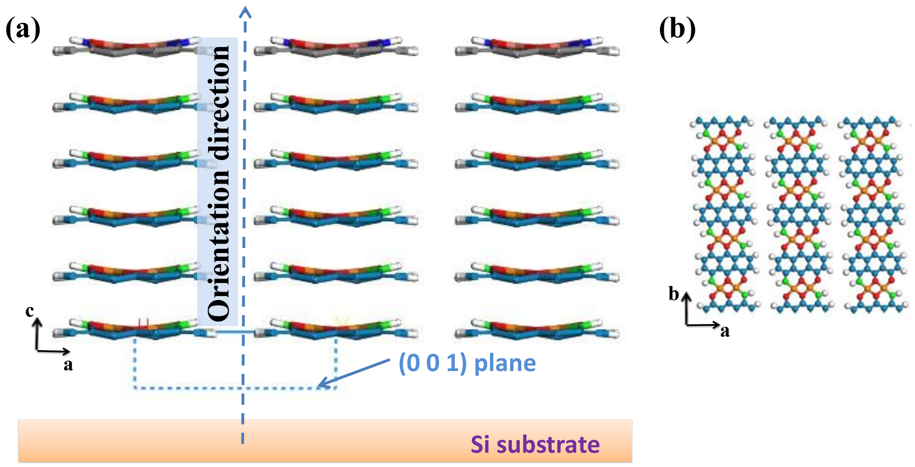

Supplementary Figure 13. Schematic diagram of orientation growth of DDA-Cu film on Si/SiO$_2$ substrate (a) and DDA-Cu structure along *ab* plane (b).

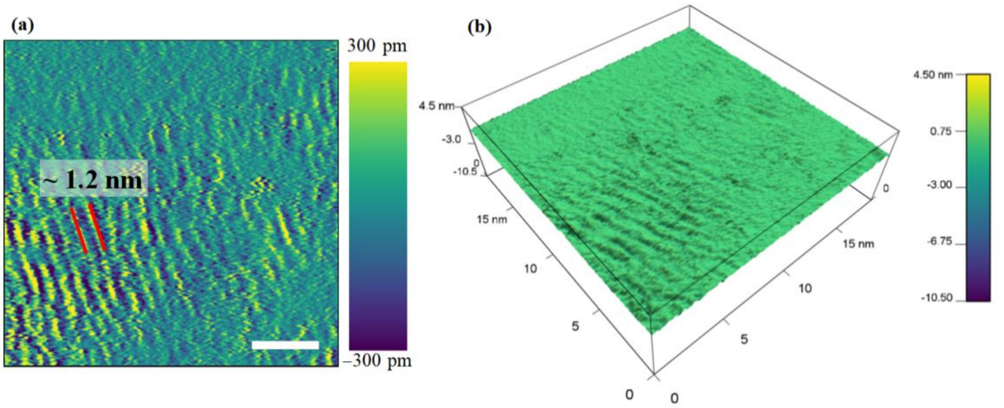

Supplementary Figure 14. HRAFM images of a DDA-Cu film. **a.** Two-dimension HRAFM image of a DDA-Cu film. **b.** Three-dimension HRAFM image of a DDA-Cu film. Scale bar 4 nm.

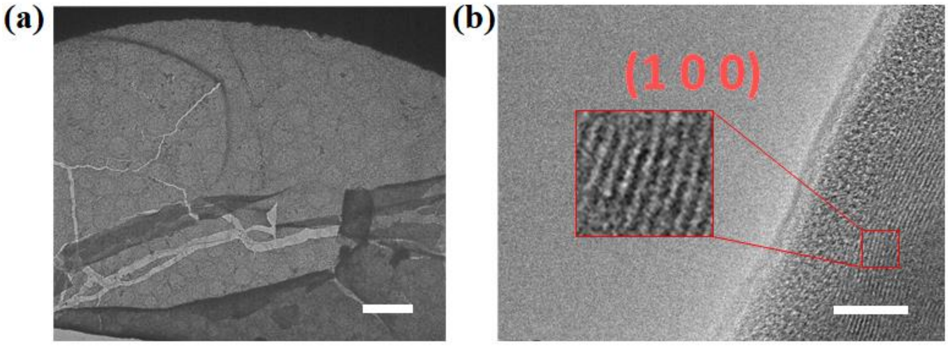

Supplementary Figure 15. TEM images of a DDA-Cu film. **a**. TEM image of a DDA-Cu film. **b**. HRTEM image of a DDA-Cu film. Scale bars 5 um (a) and 20 nm (b).

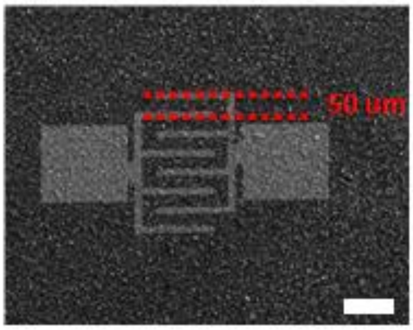

Supplementary Figure 16. SEM image of a DDA-Cu-based device. Scale bar 100 um.

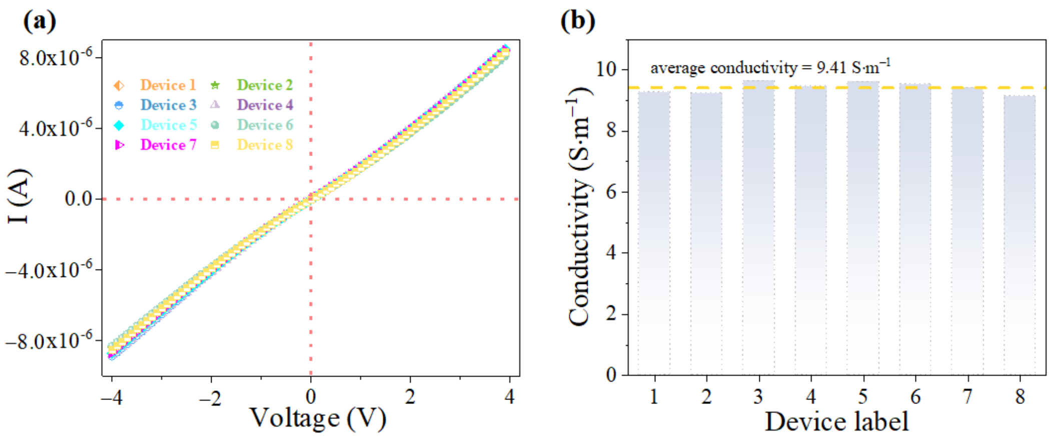

Supplementary Figure 17. a, I-V curves of DDA-Cu-based devices. **b**, The calculated values of conductivity.

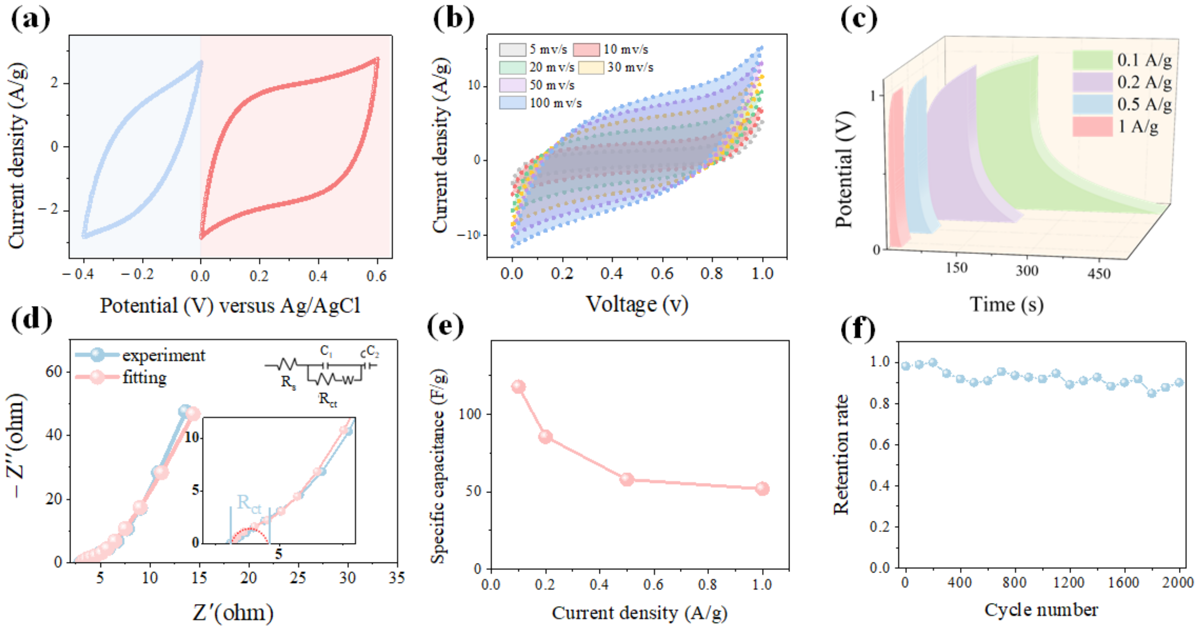

Supplementary Figure 18. Capacitance property characterization. **a**, Cyclic voltammetry curves in a three-electrode system performed at different potential range. **b**, Cyclic voltammetry curves of the symmetrical supercapacitor at different scan rates. **c**, Galvanostatic charge-discharge curves at different current density. **d**, Electrochemical impedance spectroscopy of the supercapacitor. **e**, Calculated specific gravimetric capacitance at different current density. **f**, Cycling Stability of the device. The device still retains a high retention of 92% after 2000 cycles, showing good cycle stability.

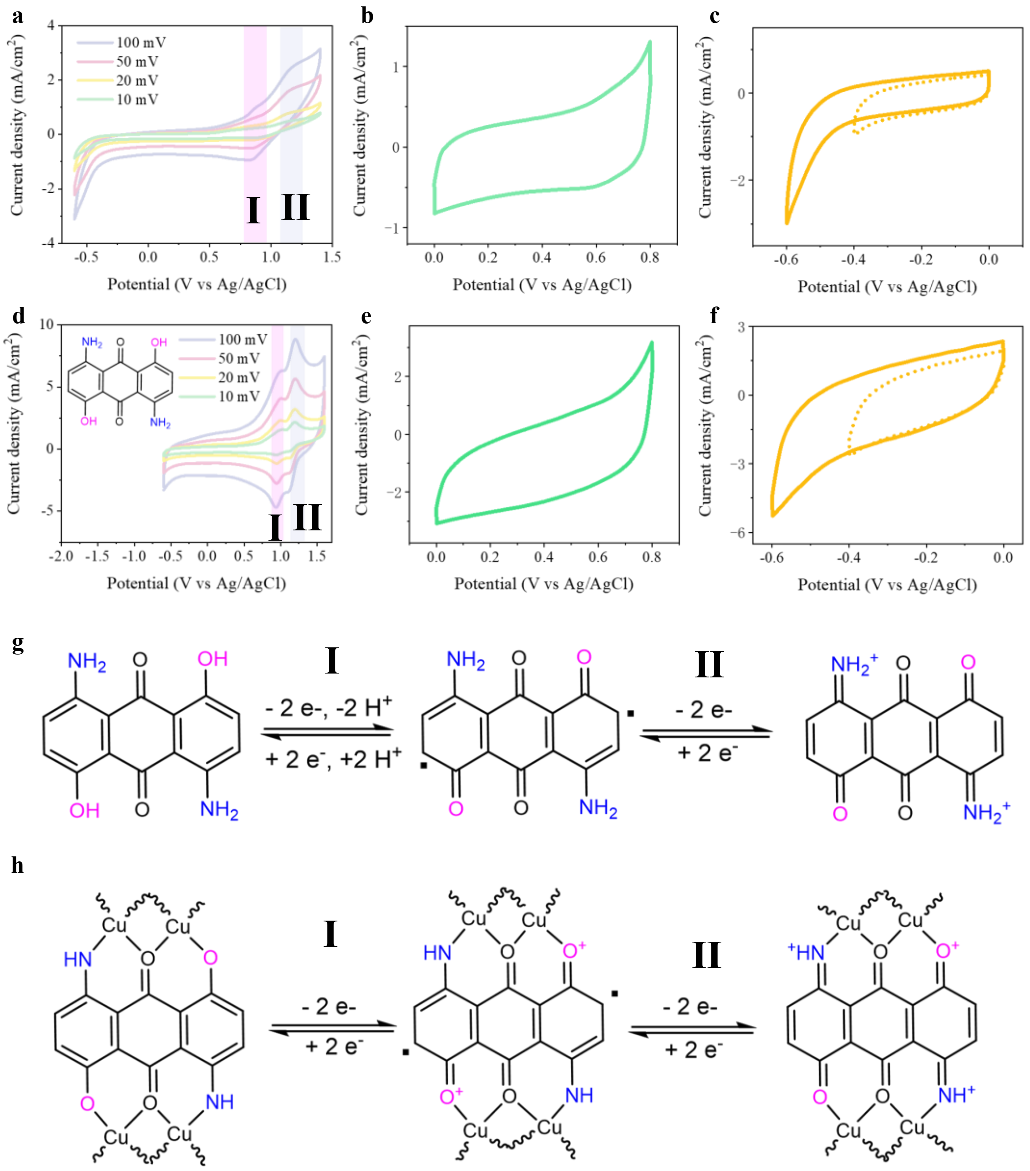

Supplementary Figure 19. CV tests and redox mechanism analysis of DDA and DAA-Cu. **a–c**, CV curves for DDA-Cu; **d–f**, CV curves for DDA; **g**,**h**, The proposed redox mechanisms for DDA and DDA-Cu, respectively. For the evaluation of DDA-Cu, we mixed 4 mg DDA-Cu with the 40 µL Naifon and 960 µL ethanol, and dropped 5 µL of the mixture to the glassy carbon electrode as the working electrode. The sweep rate of CV curves for b, c, e, f is 100 mV/s. The diameter of glassy carbon working electrode is about 3 mm.

We further investigated the capacitive properties of DDA-Cu in a wider voltage range in organic electrolytes, which are not allowed in aqueous electrolytes due to the interference of water splitting peaks. We investigated the CV curves of DDA-Cu and DDA using a three-electrode system in anhydrous CH_3CN containing [$^nBu_4N$][PF_6] (0.1 M), with Ag/AgCl as the reference electrode, Pt as

the counter electrode, and glassy carbon electrode as the working electrode. As shown in Supplementary Fig. 19a–c, two pairs of redox peaks were observed around 0.87 V and 1.1 V for DDA-Cu in the range of −0.6-1.4 V, exhibiting significant pseudocapacitive behavior. Such a large voltage window of 2 V shows that DDA-Cu may be a good candidate for a wide voltage window and high energy density supercapacitor in organic electrolyte systems[5]. When the voltage window is narrowed to −0.4-0.8 V (Supplementary Fig. 19b, c), DDA-Cu has no redox peaks. This is similar to the CV curves observed in 3 M KOH, which means double-layer capacitance behaviors. This kind of MOF-based double-layer capacitors within a certain voltage window despite the presence of redox peaks in a high voltage region has also been reported for $Ni_3(HITP)_2$[6].

To reveal the possible pseudocapacitance mechanism of DDA-Cu, we investigated the CV curves of DDA. As shown in Supplementary Fig. 19d, there are two pairs of redox peaks in DDA at approximately 0.99 V and 1.2 V, which were speculated to be responsible for the redox peaks in the DDA-Cu. Based on the previous reports of the electrochemical redox mechanisms of aniline and aromatic hydroxyl groups[7–9], the possible redox mechanisms of DDA and DDA-Cu are given in Supplementary Fig. 19g, h. There are two redox processes here for DDA/DDA-Cu, the first is the transformation of C–O to quinoid structure, and the second is the transformation of C–N to C=N. In conclusion, the above tests show that DDA-Cu exhibits pseudocapacitive behavior contributed by ligands in a high voltage region. In contrast, the aqueous DDA-Cu supercapacitors under voltage window (1 V) studied here exhibit a double-layer capacitive storage mechanism.

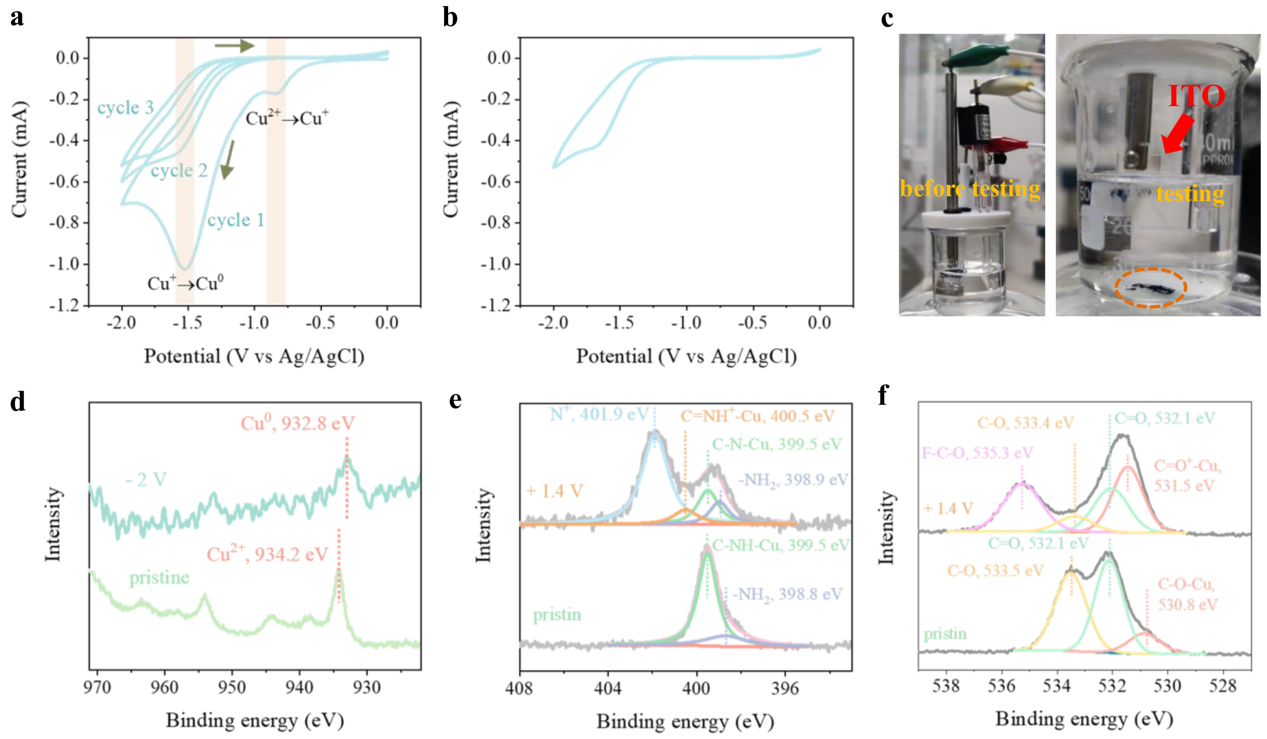

Supplementary Figure 20. a, CV curves of DDA-Cu. **b**, CV curve of background. **c**, Optical photos of the electrochemical cell before testing (left) and testing (right). **d**, Ex-situ XPS results of Cu 2p region for pristine DDA-Cu (bottom plot) and DDA-Cu charged at –2 V (top plot). **e**, **f**, Ex-situ XPS results of O1s and N1s for pristine DDA-Cu (bottom plot) and DDA-Cu charged at +1.4 V (top plot). Scan rate: 100 mV/s for a and b.

As shown in Supplementary Fig. 20a, two distinct reduction peaks were observed at –0.84 V and –1.52 V in cycle 1, assigned to Cu^{2+} to Cu^{+} process and Cu^{+} to Cu^{0} process respectively. Then we observed rapid exfoliation of DDA-Cu powder during the first scan (Supplementary Fig. 20c). And we speculated that the reduced copper would disengage from the coordination environment and destabilize the framework material, leading to exfoliation from the electrode surface. Therefore, the CV curves of cycle 2 and cycle 3 are similar to the curve for pure background (Supplementary Fig. 20b). The exfoliation was collected on conductive glass for ex-situ XPS testing. As shown in Supplementary Fig. 20d, compared to pristine DDA-Cu (Cu^{2+}, 934.2 eV), the binding energy of copper for the electrochemically treated material significantly decreases (Cu^{0}, 932.8 eV), which is consistent with the reduction peak we observed in the CV curves (Supplementary Fig. 20a). In Supplementary Fig. 20e, for N1s peak of pristine DDA-Cu, there are two kinds of N, the N that coordinates with Cu (C–NH–Cu, 399.5 eV) and the terminal amino group (–NH_2, 398.8 eV) of the framework that does not coordinate with metal. For the N1s peak of the oxidized DDA-Cu, a new

oxidation state of N appears at high binding energy (400.5 eV) assigned to the imine bond N ($C=NH^+$–Cu) and another new peak at 410.9 eV is assigned to the N^+ structure of the electrolyte [$^nBu_4N$][PF_6]. In addition, for O1s (Supplementary Fig. 20f), there are three kinds of O for pristine DDA-Cu, O coordinated with Cu (530.8 eV, C–O–Cu), and organic framework oxygen, C–O (533.5 eV) and C=O (532.1 eV) from the common contribution of uncoordinated C=O, C–OH in DDA-Cu and the ester group of acetate (may be coordinated at the end of DDA-Cu chains or crystal surfaces). For the O1s peak of the oxidized DDA-Cu, the O coordinated with Cu (C–O–Cu) is obviously moved to a high binding energy position at 531.5 eV ($C=O^+$–Cu). The peak at 535.3 eV is attributed to the F–C–O structure in Nafion adhesive. All in all, it is clear that the electrochemical oxidation significantly affects the binding energy of the heteroatoms in ligand backbone consistent with the proposed mechanism with N/O as the redox active center in Supplementary Fig. 19g, h.

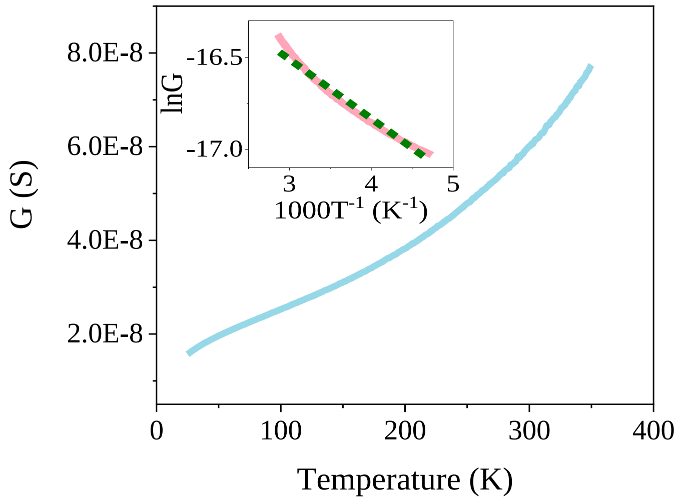

Supplementary Figure 21. Dependence of conductance with temperature (25–350 K) of DDA-Cu.

The conductance of DDA-Cu film gradually increases with the temperature increasing from 25 K to 340 K. Meanwhile a linear relationship between ln(G) and 1000/T (210–350 K) was observed indicating a typical semiconducting characteristic of the material, similar to other semiconducting MOFs[10–12].

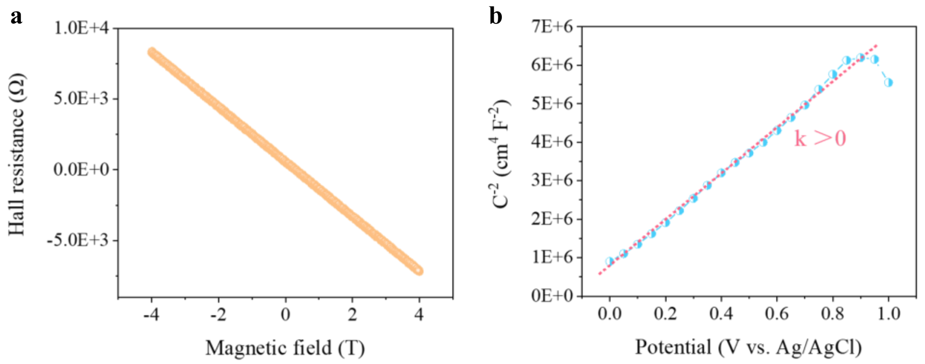

Supplementary Figure 22. Characterization of semiconductor properties. **a**, Hall effect measurements of DDA-Cu at 298 K. b, Mott–Schottky plots of DDA-Cu.

Hall effect results indicate that DDA-Cu is a typical *n*-type semiconductor (Supplementary Fig. 22a). We obtained the average carrier density and Hall mobility of 3.11×10^{17} cm^{-3} and 0.32 cm^2/Vs (Table S6), respectively, which exceeds *p*-type $K_3Fe_2[PcFe–O_8]$ MOF based on Hall effect measurements[13]. Also, we observed a positive slope in the linear region of Motto-Schotky curve (Supplementary Fig. 22b), indicating the *n*-type semiconductor of DDA-Cu. These experimental characterizations fully demonstrate that DDA-Cu is an *n*-type semiconductor. Semiconductor 2D framework materials are dominated by *p*-type, such as COFs[14]. The reported conductive semiconductor MOFs are mostly *p*-type; therefore DDA-Cu is a rare highly conductive *n*-type MOF (Table S7).

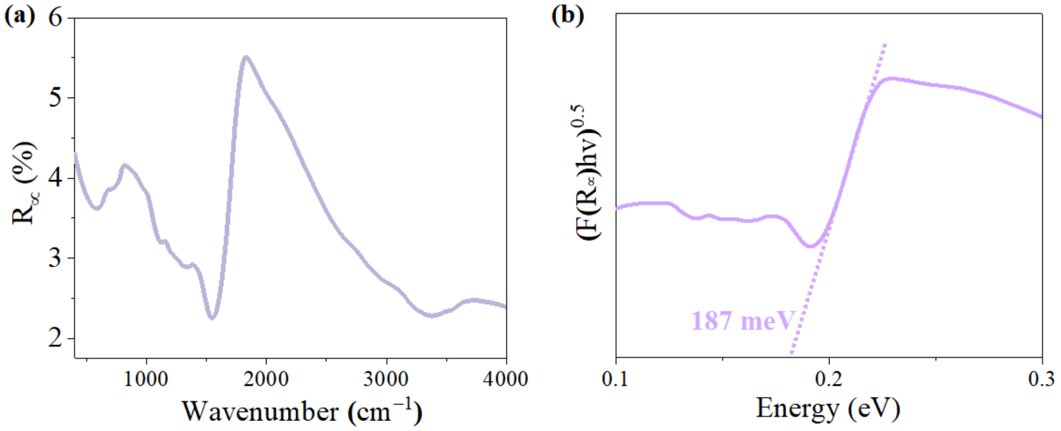

Supplementary Figure 23. a, Infrared diffuse reflection spectrum of DDA-Cu. **b**, $[F(R)\times h\upsilon]^{0.5}$ vs. hυ.

The electrical band gap was calculated by poltting the $[F(R)\times h\upsilon]^{0.5}$ vs. hυ to get the extrapolating gap value, where ν refers to frequency, h refers to Planck constant. Here, the F(R) was calculated by means of the following Kubelka–Munk (K–M) function [11]:

$$\frac{K}{S} = F(R) = \frac{(1-R)^2}{2R} \quad (1)$$

, where the K is the absorption coefficient, S is a scattering factor, R is the reflectance.

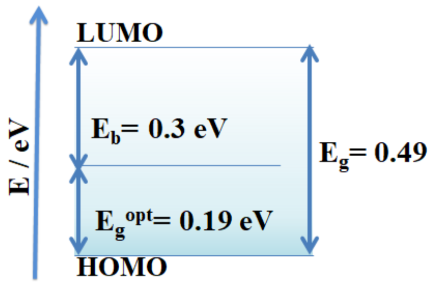

Supplementary Figure 24. Energy level diagram of DDA-Cu.

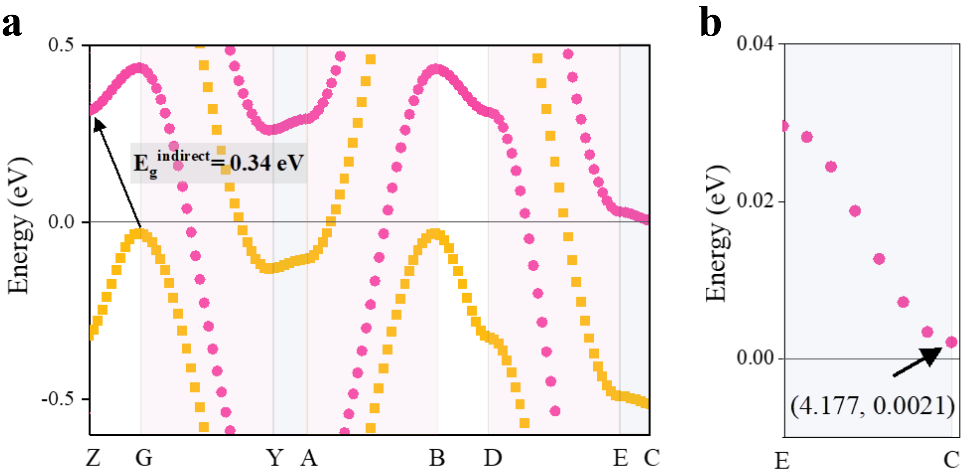

Supplementary Figure 25. Band structure of DDA-Cu. a, Band structure along Z-G-…-C paths. $E_g^{indirect}$ means indirect band gap. b, Partial enlarged picture of band structure along E-C path. The coordinate of the data point at C is (4.177, 0.0021) above the Fermi level. Therefore, DDA-Cu exhibits semiconductive features in all in-plane paths (Z-G, Y-A, B-D and E-C). However, it exhibits metallic characteristics along π-π stacking path (G-Y, A-B, and D-E). This anisotropic charge transport behavior is a manifestation of the structure-activity relationship[15,16].

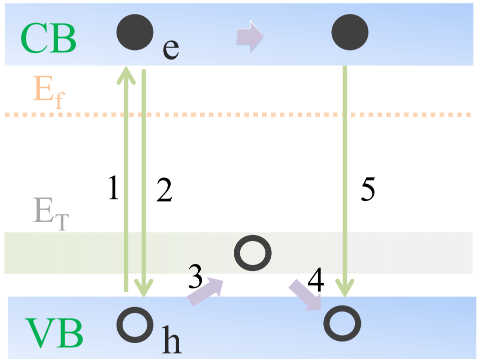

Supplementary Figure 26. The proposed mechanism for the synaptic properties of DDA-Cu. CB: conduction band; VB: valence band; h: hole; e: electron; E_f: the fermi energy; E_T: the energy level of trap states.

The conductance of the DDA-Cu film (that is proportional to carrier concentration) represents the synaptic weight of the photoelectronic device, which will change with light stimulation leading to the synaptic plasticity. When the light is on, photogenerated electrons are excited to the CB (process 1), leaving photogenerated holes in the VB. Some of the photogenerated holes are recombined with electrons immediately (process 2). And some are captured by trap states slowly (process 3; the trap states may be caused by dangling bonds at the edge of the material, etc.), resulting in the slow accumulation of photogenerated electrons in CB. Therefore, the photogenerated current gradually increases. As all trap states are filled, the electrons in CB reach a maximum concentration and the photogenerated current is gradually saturated. When the light is removed, the trap states gradually release holes (process 4) to recombine with electrons (process 5). This leads to a slow decay of the photogenerated current, illustrating the synaptic plasticity.

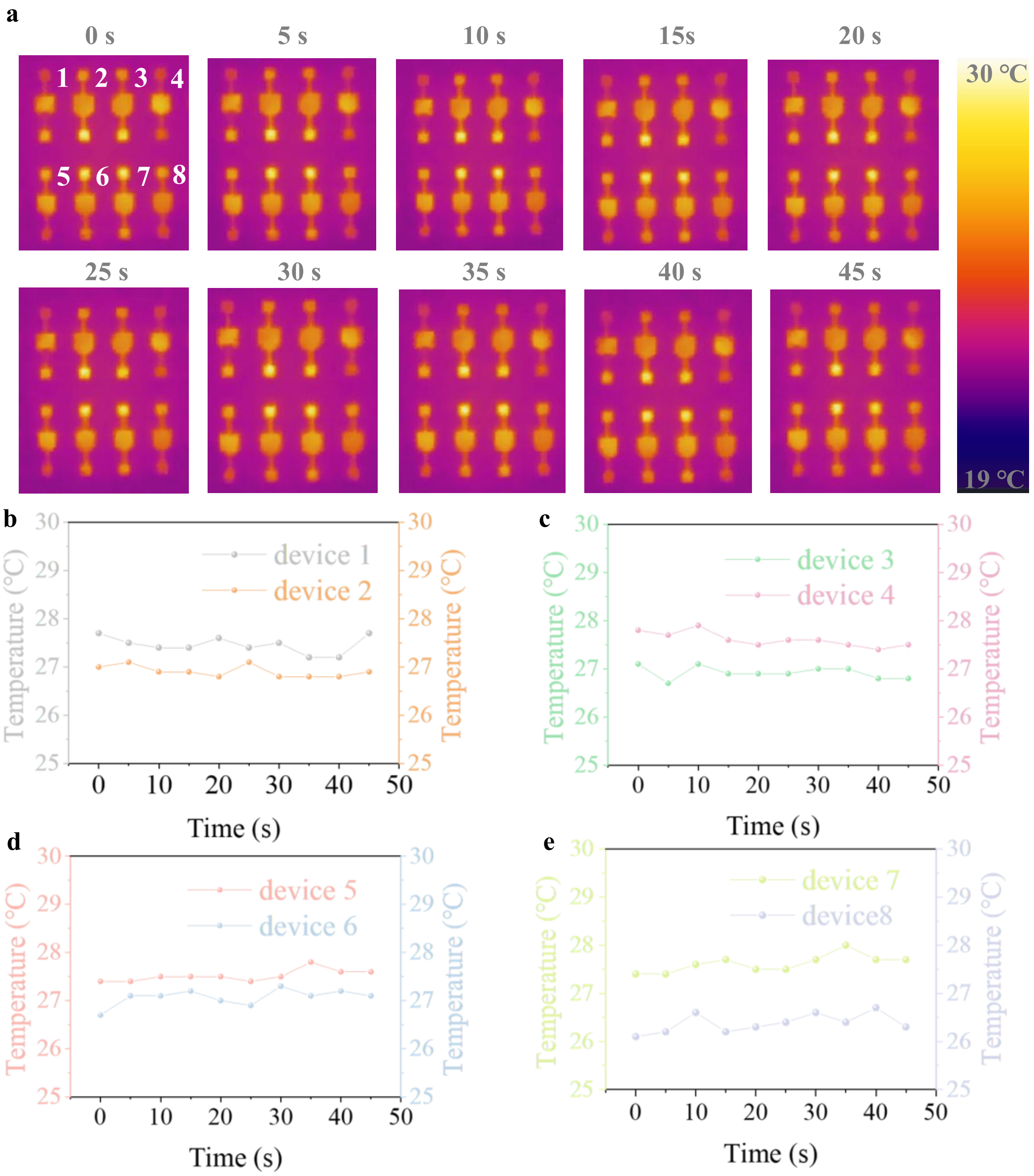

Supplementary Figure 27. a, Temperature distribution of device 1-8 under white light illumination (white light, 30 mW/cm^2); **b–e**, Temperature at the center of the device channels over time. The light is on for the first 25 s and the light removed for the last 20 seconds.

Clearly the temperature of each device fluctuates in a small range (< 1 K), and there is no obvious increase with the light on. In addition, according to the temperature dependence of conductivity, with a 1 K increase in temperature (299–301 K), the device current increases by a maximum of ~ 0.14 nA at V_{DS} = 5 V, which is significantly smaller than the measured EPSC of ~ 5 nA (based Si/SiO_2 substrate) in our manuscript. Therefore, the thermal effect of lighting can be ignored here. The similar result also has been reported before[17]. The increase in photocurrent should be attributed to the generation of photogenerated carriers in DDA-Cu (Supplementary Fig. 27).

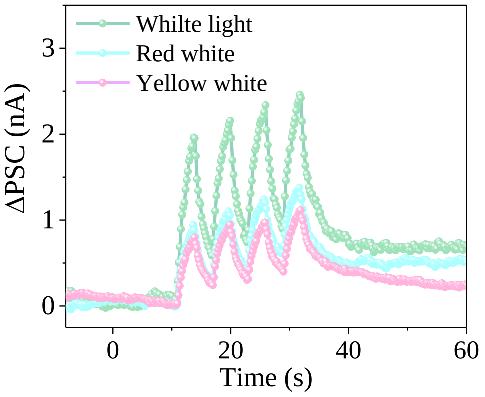

Supplementary Figure 28. The ΔPSC of devices based on Si/SiO_2 substrates stimulated by white light, red light (620–625 nm) and yellow light (590–595 nm); Excitation time is 3 s, and interval time between two light pulses is also 3 s. Source-drain voltage (V_{DS}) is 5 V.

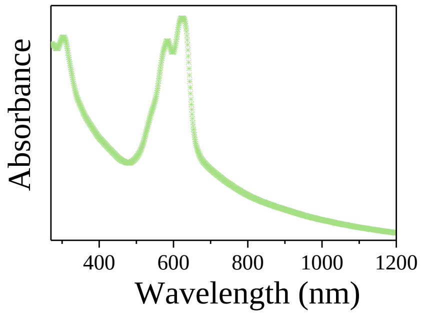

Supplementary Figure 29. UV−vis−NIR absorption spectra of DDA-Cu.

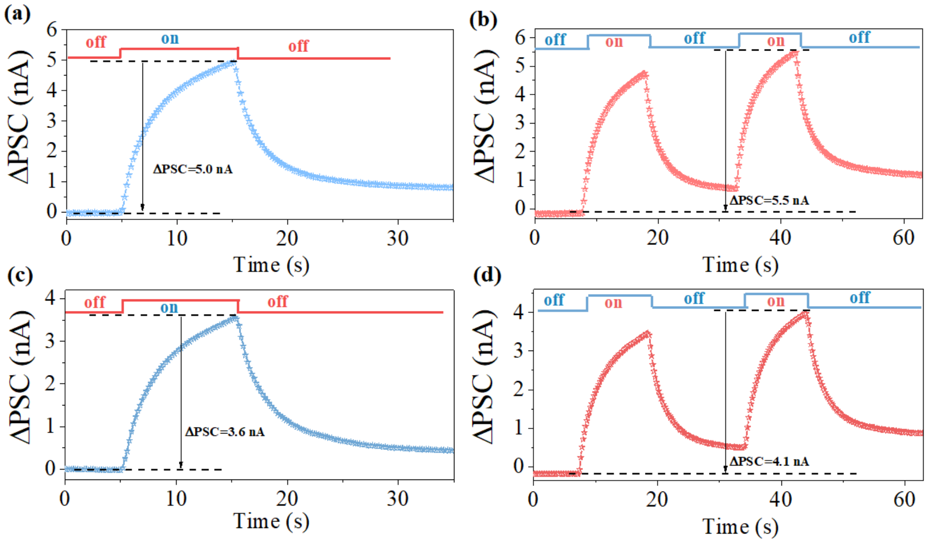

Supplementary Figure 30. a,**b**, The ΔPSC of device based on Si/SiO_2 substrate stimulated by white light (excitation time is 10 s, 20 mW/cm^2). The V_{DS} is 5 V. **c**,**d**, The ΔPSC of device based on flexible PET substrate stimulated by white light (excitation time is 10 s, interval time between two light pulses is 15 s, 20 mW/cm^2). The $V_{source\text{-}drain}$ is 5 V.

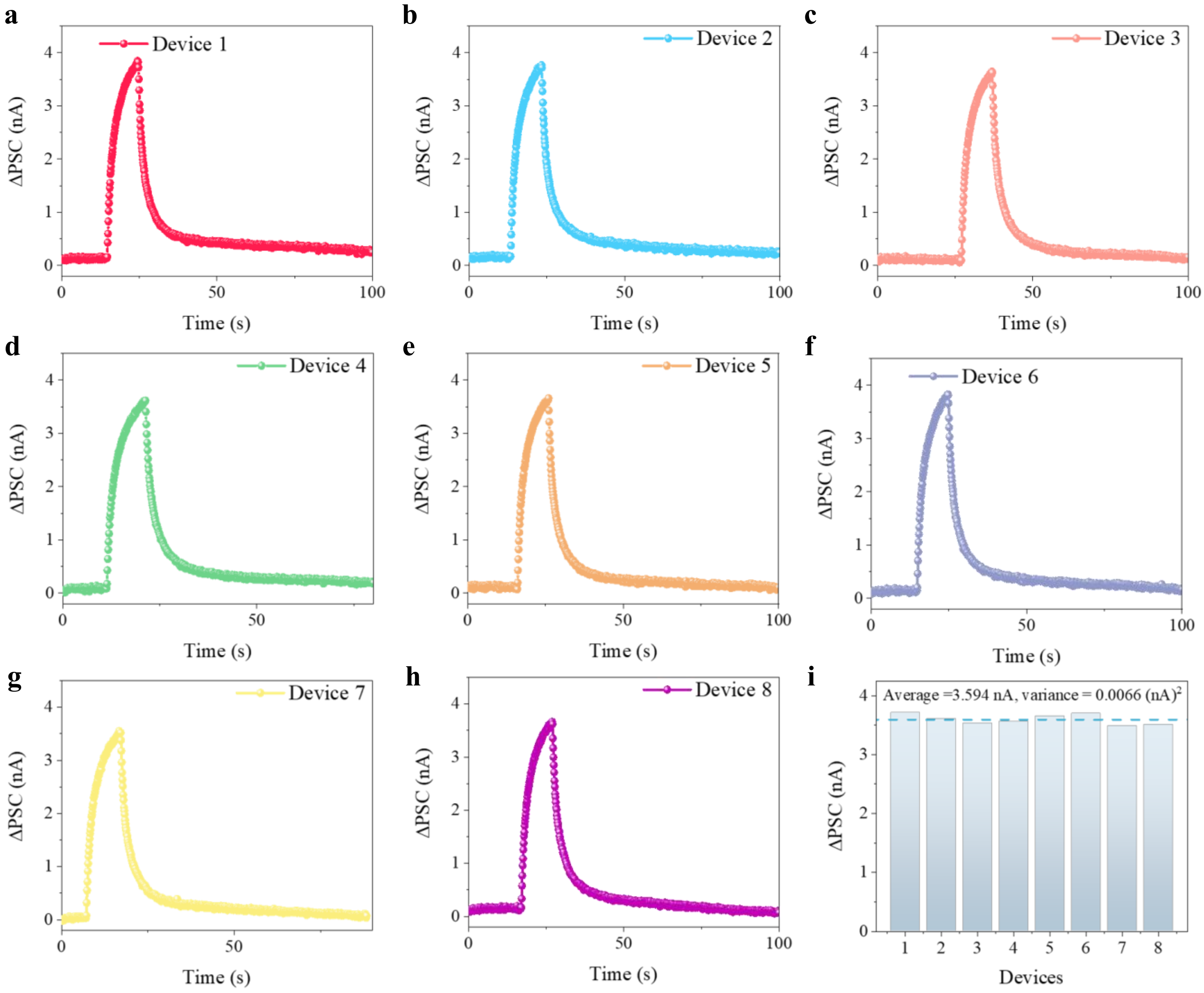

Supplementary Figure 31. a–h, Single-pulse light response signals of 8 randomly selected flexible devices. (White light, excitation time =10 s, 20 mW/cm^2) **i**, the ΔPSC values of 8 flexible devices. The low variance of 0.007 (nA)^2 shows that the light response of the devices has good reproducibility, and the optical synapse performance of different devices remains highly consistent.

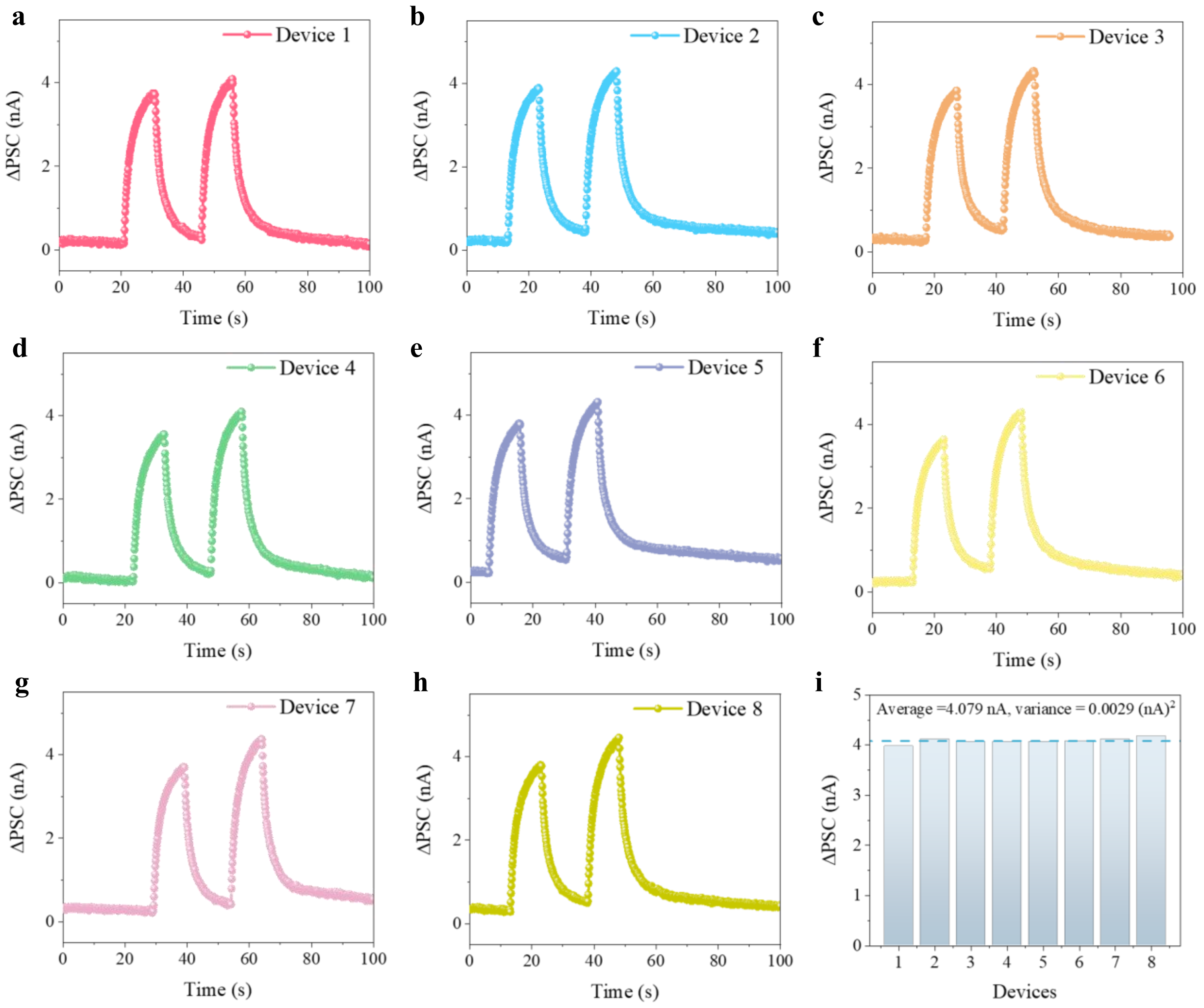

Supplementary Figure 32. a–h, Double-pulse light response signals of 8 randomly selected flexible devices. (White light, excitation time = 10 s, interval time = 15 s between two light pulses, 20 mW/cm^2) **i**, The ΔPSC values of 8 devices. The low variance of 0.003 (nA)^2 shows that the light response of the devices has good reproducibility, and the optical synapse performance of different devices remains highly consistent.

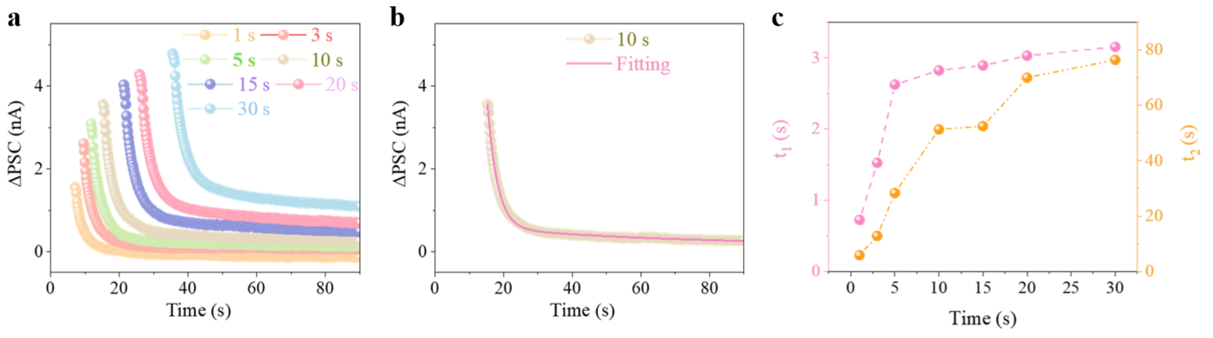

Supplementary Figure 33. **a**, Decay curves of different duration time of light. **b**, The exponential fitting of decay curve for 10 s irradiation time. **c**, Variation of time constants t_1 and t_2 with irradiation time.

All decay curves (Supplementary Fig. 34a) are fitted by a double-exponential function ($y = Ae^{(-x/t_1)} + Be^{(-x/t_2)} + C$). The constants, t_1=2.83 s and t_2= 51.30 s are obtained by fitting the decay curve with the duration time of 10 s (Supplementary Fig. 34b). The shorter one, t_1 and the longer one, t_2 usually correspond to shallower trap states and deeper trap states in material, which are closely related to the STP and LTP features, respectively, and we observed a positive correlation between the time constants and the duration time (Supplementary Fig. 34c). Those results are consistent with the previous report[18].

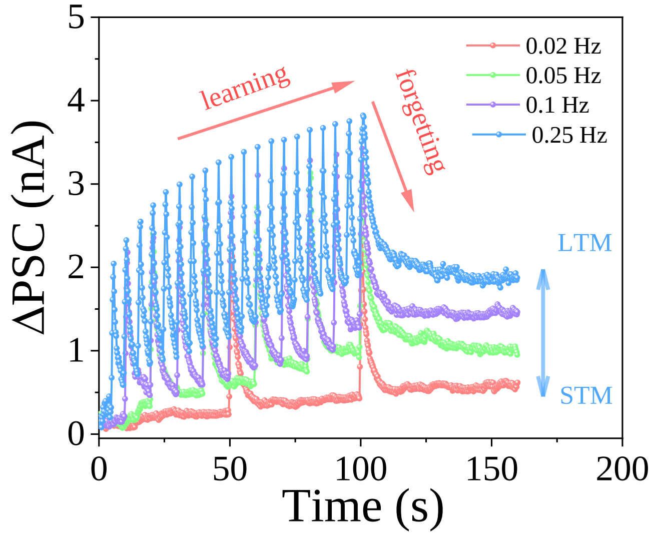

Supplementary Figure 34. The transition of STP to LTP via different light frequency.

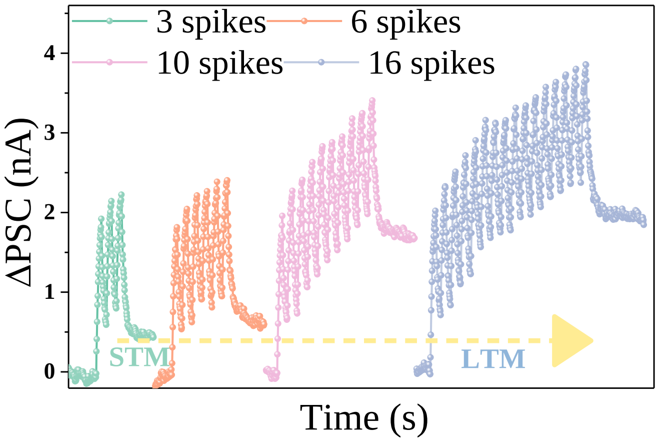

Supplementary Figure 35. The transition of STP to LTP via spike number.

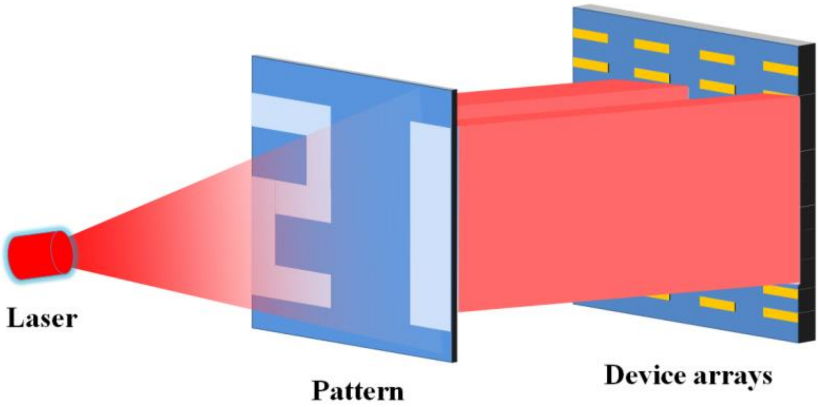

Supplementary Figure 36. Schematic diagram of pattern-recognition of devices array

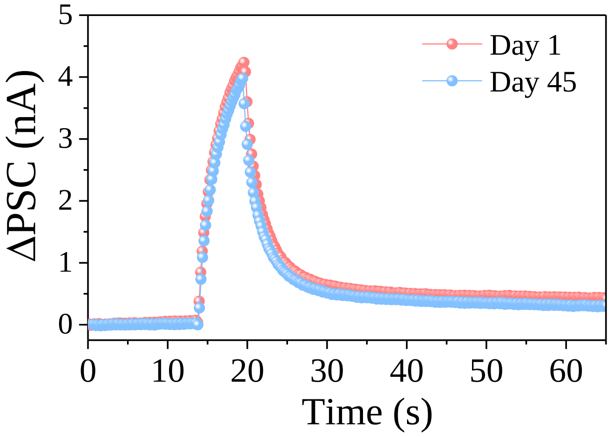

Supplementary Figure 37. The ΔPSC of a DDA-Cu-based device which is placed for 1 day and 45 days.

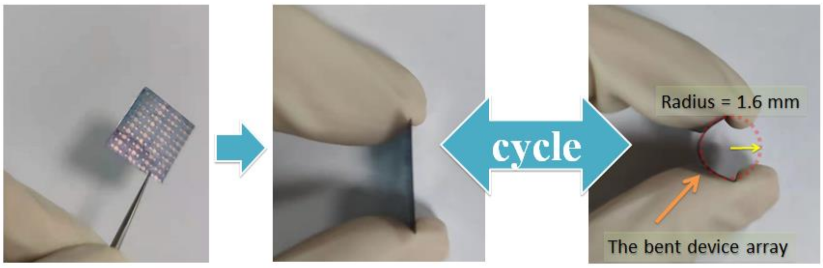

Supplementary Figure 38. The bending tests of the flexible device array. The bending radius is ~1.6 mm.

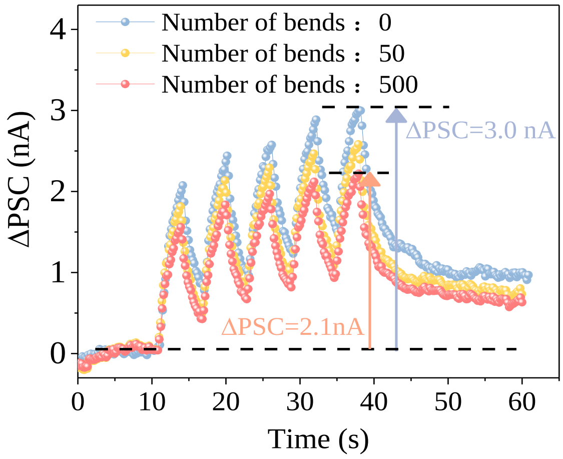

Supplementary Figure 39. Device performance comparison based on bending test

Supplementary Tables

Supplementary Table 1. Atomic coordinates of the DDA-Cu MOF.

**Space group: P2/m**			
**a = 11.8 Å, b = 7.9 Å, c = 3.2 Å, α = β = 90.0°, and γ = 87.7°**			
atom	X	Y	Z
Cu 1	0.110979997	0.502040029	0
Cu 2	0.889020026	0.497959971	0
C 1	0.110959999	0.088200003	0
C 2	0.889039993	0.911800027	0
C 3	0.218689993	0.169909999	0
C 4	0.781310022	0.830089986	0
C 5	0.683239996	0.093350001	0

C 6	0.316760004	0.906650007	0
C 7	0.785420001	0.17757	0
C 8	0.214579999	0.822430015	0
C 9	0.109070003	0.908649981	0
C 10	0.890929997	0.091350019	0
C 11	0.998640001	0.815909982	0
C 12	0.00136	0.184090018	0
C 13	0.680860043	0.921509981	0
C 14	0.319139987	0.078490019	0
N1	0.231920004	0.346509993	0
N2	0.768079996	0.653490007	0
O1	0.772859991	0.348690003	0
O2	0.227139995	0.651309967	0
O3	0.998629987	0.660969973	0
O4	0.00137	0.339030027	0
H1	0.314399987	0.395280004	0
H2	0.685600042	0.604719996	0
H3	0.604330003	0.16336	0
H4	0.395669997	0.83664	0
H5	0.599420011	0.858449996	0
H6	0.400579989	0.141550004	0

Note: This model was obtained by geometric optimization using the Forcite module of the Materials studio software package, using the Universal force field, and the electrostatic and van der Waals interaction were solved with Ewald method and atom-based method, respectively. The convergence criteria for the energy and force of the optimization process are 0.001 kcal/mol and 0.5 kcal/mol/Å, respectively. Optimization process preserved unit cell symmetry and fixed atoms in the same plane.

Supplementary Table 2. Conductivity comparison with reported 1D MOFs.

Materials	Conductivity ($S{\cdot}m^{-1}$)	Method	State	Reference
DDA-Cu	9.4	Two-probe	film	This work
**1**	3×10^{-9}	Two- probe	pellet	19
**2**	5×10^{-8}	Two- probe	pellet	19
$Cu(fum)(4\text{-}phpy)_2$	6×10^{-4}	Two- probe	film	20
Cd(nip)(4-Clpy)	8.5×10^{-4}	Two- probe	film	21
Cd(nip)(4-Brpy)	1×10^{-3}	Two- probe	film	21
Cd(nip)(4-Phpy)	1.4×10^{-3}	Two- probe	film	21
Co(adc)(4-ppy)	2.6×10^{-5}	Two- probe	film	22
Co(adc)(4-bppy)	2.5×10^{-6}	Two- probe	film	22
PMC-1	$1.0\text{-}3.3\times10^{-1}$	Two- probe	single crystal	23
$[Cu\text{-}(abpy)]_n$	9.3	Two- probe	pellet	24
$[Pt–Rh–Pt_4–Rh–Pt–Cl–]_n$	3.5×10^{-4}	Four- probe	single crystal	25
$[Ag(3\text{-}cyanopyridine)_2(NO_3)]_n$	1×10^{-9}	Two- probe	pellet	26
$[Ag(dpbd)(ClO_4)]$	2.1×10^{-5}	Two- probe	single crystal	27
$[Ag_2(bmsb)(ClO_4)_2]$	1×10^{-4}	Two- probe	single crystal	28
$[Cu_5(m\text{-}4\text{-}SpyH)_7(m\text{-}I)I_4]_n$	6.8×10^{-6}	Two- probe	single crystal	29
$\{[Cu_2(m\text{-}I)(m\text{-}4\text{-}Spy)_3]I\}_n$	2.7×10^{-7}	Two- probe	single crystal	29
$[Rh_2(acam)_4Br]_n$	2×10^{-5}	Two- probe	pellet	30
$[Rh_2(acam)_4I]_n$	2×10^{-5}	Two- probe	pellet	30

Note: Solvent molecules are omitted in the formulas for reference 3 and 4. All values are obtained at 297 K.

Supplementary Table 3. Conductivity comparison of conductive COFs/MOFs films.

Materials	Conductivity ($S{\cdot}m^{-1}$)	Method	Temperature	Reference
DDA-Cu	9.4	Two-probe	297 K	This work
I_2-doped PAE-PcCu	0.2	Two-probe	297 K	[31]
$Cu_3(HHTP)_2$	8.7	Two-probe	297 K	[32]
I_2-doped $Cu(Ni(pdt)_2)$	0.01	Two-probe	297 K	[33]
TCNQ@$Cu_3(BTC)_2$	7	Four-probe	297 K	[34]
2D $Pd_3(BHT)_2$	2.8	Four-probe	297 K	[35]
$Cu[Ni(pdt)_2]$	1×10^{-6}	Two-probe	297 K	[33]

Supplementary Table 4. C_g comparison based solid-state supercapacitors.

Materials	Cg (F/g)	Rate (A/g)	Class	Reference
DDA-Cu	118	0.1	MOFs	This work
$Ni_3(HITP)_2$	111	0.05	MOFs	[6]
Ni-MOF	96.7	1	MOFs	[36]
Activated-CC	4.4	(10 mV/s)	Activated carbons	[37]
CCG@SSF	27.2±0.6	4	Activated carbons	[38]
Carbon SSCs	101	0.125	Activated carbons	[39]
SWCNT film	35	30	SWCNT	[40]
CNT/PANI	31.4	1	SWCNT	[41]
Carbon nanotube	7.3	0.25	SWCNT	[42]
GF@3D-G	30	1	Graphene	[43]
PG-CC	79.19	0.5	Graphene	[44]
aRGO20	53	1	Graphene	[45]

Supplementary Table 5. Performance summary of optoelectronic synapse devices.

Materials	Device	Wavelength (nm)	PPF	Imaging function	Flexible devices	Ref.
DDA-Cu	2T	White light, 620, 590	121 %, Δ t = 1 s	√	√	This work
Au/LSNO/Au	2T	365, 450, 520, 730	≈ 138 %, Δ t = 1 s	√	/	46
ITO/Nb: $SrTiO_3$	2T	459, 528, 630	≈ 145 %, Δ t = 0.5 s	√	/	47
IGZO	2T	380–385	/	/	/	48
Pd/MoO_x/ITO	2T	365	/	√	/	49
$(PEA)_2SnI_4$	2T	470, 590, 660	/	/	√	50
MoS_2	2T	310	120 %, Δ t = 1 s	/	/	51
CS: pentacene	3T	365–660	≈ 102–130 %	/	√	52
CS: Dif-TES-ADT	3T	White light	≈ 240 %, Δ t = 0.3 s	√	√	53
C_3N_4	3T	350–400	≈ 120 %, Δ t = 0.04 s	/	/	54
$CsPbBr_3$ QDs	3T	660, 520, 450	107 %, 112 %, 117 %	/	/	55
a-IGZO/CdS	3T	Green light	119 %, Δ t = 0.3 s	√	/	56

Note: "2T" and "3T" represent "two-terminal" and "three-terminal", respectively. "Δ t" means "interval time between light spikes". "√" and "/" represent "present" and "no present", respectively.

The devices in this work display a high PPF value (121 %) with achieving multi-wavelength synaptic response similar to those reported for two-terminal/three-terminal devices recently. Compared with traditional multi-layer composite devices, our devices achieve similar optical synaptic functions using only one MOF material as the active layer with the advantages of simpler device fabrication and avoiding complex interfacial compatibility problems. More importantly, the imaging function and flexibility characteristics exhibited by our device indicate its important potential in flexible optoelectronic devices in the future.

Supplementary Table 6. Hall effect results of three devices.

Sample	Carrier	Carrier density	Hall coefficient	Carrier mobility
1	*n*-type	3.22726E17 cm^{-3}	–19.33531 cm^3/C	0.30528 cm^2/Vs
2	*n*-type	2.99617E17 cm^{-3}	–20.82657 cm^3/C	0.31635 cm^2/Vs
3	*n*-type	3.09418E17 cm^{-3}	–20.16688 cm^3/C	0.32649 cm^2/Vs
Average value	/	3.10587E17 cm^{-3}	–20.10959 cm^3/C	0.31604 cm^2/Vs

Supplementary Table 7. Type of semiconductor MOFs.

Materials	Type	Technology	Ref.
Cu-CAT-1	*p*	FETs	57
$K_3Fe_2[PcFe–O_8]$	*p*	Hall effect	13
$Fe_3(THT)_2(NH_4)_3$	*p*	Hall effect	10
$Ni_3(HITP)_2$	*p*	FETs	58
$(ZnC_2O_3H_2)_n$	*p*	Mott−Schottky plot	59
NTU-9	*p*	Mott−Schottky plot	60
Ni-HAB	*n*	FETs	61
$[Co_3(THT)_2]^{3-}$	/	/	62
FeTHT	/	/	63

Supplementary References

(1) Pham, H. T. B., *et al.*, Imparting functionality and enhanced surface area to a 2D electrically conductive mof via macrocyclic linker. *J. Am. Chem. Soc.* **144,** 10615–10621 (2022).
(2) Feng, D., *et al.*, Robust and conductive two-dimensional metal−organic frameworks with exceptionally high volumetric and areal capacitance. *Nat. Energy* **3,** 30–36 (2018).
(3) Phua, E. J. H., *et al.*, Oxidation-promoted interfacial synthesis of redox-active bis(diimino)nickel nanosheet. *Chem. Lett.* **47,** 126–129 (2018).
(4) Li, L., et al., Discrimination of xylene isomers in a stacked coordination polymer. *Science* **377**, 335–339 (2022).
(5) Zhang, P., *et al.*, Dual-redox-sites enable two-dimensional conjugated metal-organic frameworks with large pseudocapacitance and wide potential window. *J. Am. Chem. Soc.* **143,** 10168–10176 (2021).
(6) Sheberla, D., *et al.*, Conductive mof electrodes for stable supercapacitors with high areal capacitance. *Nat. Mater.* **16,** 220–224 (2017).
(7) Yang, Z., *et al.*, Arylamine-linked 2D covalent organic frameworks for efficient pseudocapacitive

energy storage. *Angew. Chem. Int. Ed. Engl.* **60,** 20754–20759 (2021).
(8) Chandra, S., *et al.*, Molecular level control of the capacitance of two-dimensional covalent organic frameworks: Role of hydrogen bonding in energy storage materials. *Chem. Mater.* **29,** 2074–2080 (2017).
(9) Scotto, J.; Marmisollé, W. A.; Posadas, D., About the capacitive currents in conducting polymers: The case of polyaniline. *J. Solid State Electrochem.* **23,** 1947–1965 (2019).
(10) Dong, R., *et al.*, High-mobility band-like charge transport in a semiconducting two-dimensional metal-organic framework. *Nat. Mater.* **17,** 1027–1032 (2018).
(11) Pathak, A., *et al.*, Integration of a $(\text{-Cu-S-})_n$ plane in a metal-organic framework affords high electrical conductivity. *Nat. Commun.* **10,** 1721 (2019).
(12) Xie, L. S., *et al.*, Tunable mixed-valence doping toward record electrical conductivity in a three-dimensional metal-organic framework. *J. Am. Chem. Soc.* **140,** 7411–7414 (2018).
(13) Yang, C., *et al.*, A semiconducting layered metal-organic framework magnet. *Nat. Commun.* **10,** 3260 (2019).
(14) Wang, S., *et al.*, Semiconductive covalent organic frameworks: Structural design, synthesis, and application. *Small Struct.* **1,** 2000021 (2020).
(15) Nyakuchena, J., *et al.*, Direct evidence of photoinduced charge transport mechanism in 2D conductive metal organic frameworks. *J. Am. Chem. Soc.* **142,** 21050–21058 (2020).
(16) Skorupskii, G., *et al.*, Efficient and tunable one-dimensional charge transport in layered lanthanide metal-organic frameworks. *Nat. Chem.* **12,** 131–136 (2020).
(17) Tan, H., *et al.*, Broadband optoelectronic synaptic devices based on silicon nanocrystals for neuromorphic computing. *Nano Energy* **52,** 422–430 (2018).
(18) Sun, Y., *et al.*, Photoelectric synaptic plasticity realized by 2D perovskite. *Adv. Funct. Mater.* **29,** 1902538 (2019).
(19) Horwitz, N. E., *et al.*, Redox-active 1D coordination polymers of iron-sulfur clusters. *J. Am. Chem. Soc.* **141,** 3940–3951 (2019).
(20) Ahmed, F., *et al.*, Synthesis and structural characterization of a Cu(II)-based 1D coordination polymer and its application in schottky devices. *New J. Chem.* **41,** 11317–11323 (2017).
(21) Dutta, B.; Dey, A.; Sinha, C.; Ray, P. P.; Mir, M. H., Tuning of the para-position of pyridyl ligands impacts the electrical properties of a series of Cd(II) ladder polymers. *Dalton Trans.* **48,** 11259–11267 (2019).
(22) Ahmed, F., *et al.*, Linear dicarboxylate-based pyridyl-appended cobalt(II) coordination polymers in search of opto-electronic properties. *New J. Chem.* **44,** 9004–9009 (2020).
(23) Qu, L., *et al.*, Porous molecular conductor: Electrochemical fabrication of through-space conduction pathways among linear coordination polymers. *J. Am. Chem. Soc.* **141,** 6802–6806 (2019).
(24) Clayman, N. E., *et al.*, Carving out pores in redox-active one-dimensional coordination polymers. *Angew. Chem. Int. Ed. Engl.* **57,** 14585–14588 (2018).
(25) Uemura, K., *et al.*, Paramagnetic platinum-rhodium octamers bridged by halogen ions to afford a quasi-1D system. *Angew. Chem. Int. Ed. Engl.* **44,** 5459–5464 (2005).
(26) Lin, P., *et al.*, New 1- and 2-dimensional polymeric structures of cyanopyridine complexes of Ag(I) and Cu(I). *Inorg. Chem.* **43,** 181–188 (2004).
(27) Zhong, J. C., *et al.*, Spiral, herringbone, and triple-decker silver(I) complexes of benzopyrene derivatives assembled through eta(2)-coordination. *Inorg. Chem.* **40,** 3191–3199 (2001).

(28) Liu, S. Q., *et al.*, Silver(I) coordination polymers of fluorescent oligo(phenylenevinylene) with π-π stackings: Luminescence and conductivity. *Inorg. Chem.* **44,** 1031–1036 (2005).
(29) Chen, Y., *et al.*, Solvothermal stepwise formation of Cu/I/S-based semiconductors from a three-dimensional net to one-dimensional chains. *Cryst. Growth Des.* **9,** 4963–4968 (2009).
(30) Yang, Z.; Ebihara, M.; Kawamura, T.; Okubo, T.; Mitani, T., An infinite zigzag chain of the halide-bridged tetrakis(acetamidato)dirhodium cation radical. *Inorg. Chim. Acta* **321,** 97–106 (2001).
(31) Yang, C., *et al.*, Chemically stable polyarylether-based metallophthalocyanine frameworks with high carrier mobilities for capacitive energy storage. *J. Am. Chem. Soc.* **143,** 17701–17707 (2021).
(32) Liu, Y., *et al.*, Electrochemical synthesis of large area two-dimensional metal-organic framework films on copper anodes. *Angew. Chem. Int. Ed. Engl.* **60,** 2887–2891 (2021).
(33) Kobayashi, Y.; Jacobs, B.; Allendorf, M. D.; Long, J. R., Conductivity, doping, and redox chemistry of a microporous dithiolene-based metal−organic framework. *Chem. Mater.* **22,** 4120–4122 (2010).
(34) Talin, A. A., *et al.*, Tunable electrical conductivity in metal-organic framework thin-film devices. *Science* **343,** 66–69 (2014).
(35) Pal, T., *et al.*, Interfacial synthesis of electrically conducting palladium bis(dithiolene) complex nanosheet. *ChemPlusChem* **80,** 1255–1258 (2015).
(36) Jiao, Y., *et al.*, Layered nickel metal–organic framework for high performance alkaline battery-supercapacitor hybrid devices. *J. Mater. Chem. A* **4,** 13344–13351 (2016).
(37) Wang, G., *et al.*, Solid-state supercapacitor based on activated carbon cloths exhibits excellent rate capability. *Adv. Mater.* **26,** 2676–2682, 2615 (2014).
(38) Yu, J., *et al.*, Metallic fabrics as the current collector for high-performance graphene-based flexible solid-state supercapacitor. *ACS Appl. Mater. Inter.* **8,** 4724–4729 (2016).
(39) Huang, C.-W., *et al.*, Gel electrolyte derived from poly(ethylene glycol) blending poly(acrylonitrile) applicable to roll-to-roll assembly of electric double layer capacitors. *Adv. Funct. Mater.* **22,** 4677–4685 (2012).
(40) Kaempgen, M.; Chan, C. K.; Ma, J.; Cui, Y.; Gruner, G., Printable thin film supercapacitors using single-walled carbon nanotubes. *Nano Lett.* **9,** 1872–1876 (2009).
(41) Meng, C.; Liu, C.; Chen, L.; Hu, C.; Fan, S., Highly flexible and all-solid-state paperlike polymer supercapacitors. *Nano Lett.* **10,** 4025–4031 (2010).
(42) Chen, T.; Peng, H.; Durstock, M.; Dai, L., High-performance transparent and stretchable all-solid supercapacitors based on highly aligned carbon nanotube sheets. *Sci. Rep.* **4,** 3612 (2014).
(43) Meng, Y., *et al.*, All-graphene core-sheath microfibers for all-solid-state, stretchable fibriform supercapacitors and wearable electronic textiles. *Adv. Mater.* **25,** 2326–2331 (2013).
(44) Wang, S.; Pei, B.; Zhao, X.; Dryfe, R. A. W., Highly porous graphene on carbon cloth as advanced electrodes for flexible all-solid-state supercapacitors. *Nano Energy* **2,** 530–536 (2013).
(45) Chang, H.-W., *et al.*, Electrochemically activated reduced graphene oxide used as solid-state symmetric supercapacitor: An x-ray absorption spectroscopic investigation. *J. Phys. Chem. C* **120,** 22134–22141 (2016).
(46) Zhao, L., *et al.*, An artificial optoelectronic synapse based on a photoelectric memcapacitor. *Adv. Electron. Mater.* **6,** 1900858 (2019).
(47) Gao, S., *et al.*, An oxide schottky junction artificial optoelectronic synapse. *ACS Nano* **13,** 2634–2642 (2019).
(48) Lee, M., *et al.*, Brain-inspired photonic neuromorphic devices using photodynamic amorphous

oxide semiconductors and their persistent photoconductivity. *Adv. Mater.* **29,** 1700951 (2017).
(49) Zhou, F., *et al.*, Optoelectronic resistive random access memory for neuromorphic vision sensors. *Nat. Nanotechnol.* **14,** 776–782 (2019).
(50) Qian, L., *et al.*, A lead-free two-dimensional perovskite for a high-performance flexible photoconductor and a light-stimulated synaptic device. *Nanoscale* **10,** 6837–6843 (2018).
(51) Wang, S., *et al.*, A MoS_2/PTCDA hybrid heterojunction synapse with efficient photoelectric dual modulation and versatility. *Adv. Mater.* **31,** e1806227 (2019).
(52) Wang, Y., *et al.*, Synergies of electrochemical metallization and valance change in all-inorganic perovskite quantum dots for resistive switching. *Adv. Mater.* **30,** e1800327 (2018).
(53) Deng, W., *et al.*, Organic molecular crystal-based photosynaptic devices for an artificial visual-perception system. *NPG Asia Mater.* **11,** 77 (2019).
(54) Park, H. L., *et al.*, Retina-inspired carbon nitride-based photonic synapses for selective detection of UV light. *Adv. Mater.* **32,** e1906899 (2020).
(55) Wang, Y., *et al.*, Photonic synapses based on inorganic perovskite quantum dots for neuromorphic computing. *Adv. Mater.* **30,** e1802883 (2018).
(56) Kwon, S. M., *et al.*, Large-area pixelized optoelectronic neuromorphic devices with multispectral light-modulated bidirectional synaptic circuits. *Adv. Mater.* **33,** e2105017 (2021).
(57) Rubio-Gimenez, V., *et al.*, Bottom-up fabrication of semiconductive metal-organic framework ultrathin films. *Adv. Mater.* **30,** 1704291 (2018).
(58) Wu, G.; Huang, J.; Zang, Y.; He, J.; Xu, G., Porous field-effect transistors based on a semiconductive metal-organic framework. *J. Am. Chem. Soc.* **139,** 1360–1363 (2017).
(59) Shang, C., *et al.*, A *p*-type zinc-based metal-organic framework. *Inorg. Chem.* **56,** 6208–6213 (2017).
(60) Gao, J., *et al.*, A *p*-type Ti(IV)-based metal-organic framework with visible-light photo-response. *Chem. Commun.* **50,** 3786–3788 (2014).
(61) Lahiri, N.; Lotfizadeh, N.; Tsuchikawa, R.; Deshpande, V. V.; Louie, J., Hexaaminobenzene as a building block for a family of 2D coordination polymers. *J. Am. Chem. Soc.* **139,** 19–22 (2017).
(62) Clough, A. J., *et al.*, Metallic conductivity in a two-dimensional cobalt dithiolene metal-organic framework. *J. Am. Chem. Soc.* **139,** 10863–10867 (2017).
(63) Clough, A. J., *et al.*, Room temperature metallic conductivity in a metal-organic framework induced by oxidation. *J. Am. Chem. Soc.* **141,** 16323–16330 (2019).
